# Author Correction: A cattle graph genome incorporating global breed diversity

**DOI:** 10.1038/s41467-022-30372-x

**Published:** 2022-05-23

**Authors:** A. Talenti, J. Powell, J. D. Hemmink, E. A. J. Cook, D. Wragg, S. Jayaraman, E. Paxton, C. Ezeasor, E. T. Obishakin, E. R. Agusi, A. Tijjani, W. Amanyire, D. Muhanguzi, K. Marshall, A. Fisch, B. R. Ferreira, A. Qasim, U. Chaudhry, P. Wiener, P. Toye, L. J. Morrison, T. Connelley, J. G. D. Prendergast

**Affiliations:** 1 The Roslin Institute, Royal (Dick) School of Veterinary Studies, University of Edinburgh, Easter Bush Campus, Midlothian, EH25 9RG UK; 2 The International Livestock Research Institute, PO Box 30709 Nairobi, Kenya; 3 Centre for Tropical Livestock Genetics and Health, Easter Bush, Midlothian, EH25 9RG UK; 4 Centre for Tropical Livestock Genetics and Health, ILRI Kenya, Nairobi, 30709-00100 Kenya; 5 Department of Veterinary Pathology and Microbiology, University of Nigeria, Nsukka, Enugu State Nigeria; 6 Biotechnology Division, National Veterinary Research Institute, Vom, Plateau State Nigeria; 7 Biomedical Research Centre, Ghent University Global Campus, Songdo, Incheon, South Korea; 8 International Livestock Research Institute (ILRI) PO, 5689 Addis Ababa, Ethiopia; 9 Centre for Tropical Livestock Genetics and Health (CTLGH), ILRI Ethiopia, PO Box 5689 Addis Ababa, Ethiopia; 10 Ribeirão Preto College of Nursing, University of Sao Paulo, Ribeirão Preto, SP Brazil; 11 Faculty of Veterinary and Animal Sciences, Gomal University, Dera Ismail Khan, Pakistan

**Keywords:** Animal breeding, Genomics, Animal breeding, Genomics

Correction to: *Nature Communications* 10.1038/s41467-022-28605-0, published online 17 February 2022.

The original version of this Article omitted from the author list the 12^th^ and 13^th^ authors Dennis Muhanguzi and Wilson Amanyire, who are from the ‘School of Biosecurity, Biotechnology and Laboratory Sciences (SBLS), College of Veterinary Medicine, Animal Resources and Biosecurity, Makerere University, P.O Box 7062, Kampala, Uganda’. Consequently, the final sentence of the Author Contributions incorrectly read ‘D.W., P.T., E.A.J.C., C.E., E.T.O., E.R.A., A. Tijjani, K.M., A.F., B.R.F., A.Q., U.C. and P.W. provided samples and expertise for the studies’. This has been replaced with ‘D.W., P.T., W.A., D.M., E.A.J.C., C.E., E.T.O., E.R.A., A. Tijjani, K.M., A.F., B.R.F., A.Q., U.C. and P.W. provided samples and expertise for the studies’. This has been corrected in both the PDF and HTML versions of the Article.

