# Supplementary material for: A cattle graph genome incorporating global breed diversity
Source: Nat Commun. 2022 Feb 17;13:910. doi: 10.1038/s41467-022-28605-0 (PMC8854726; doi:10.1038/s41467-022-28605-0)
Supplement: Supplementary file 1 — Supplementary Information [file 41467_2022_28605_MOESM1_ESM.pdf]

A cattle graph genome incorporating global breed diversity

Talenti et al.

This PDF file includes:

1) Supplementary Figure 1-4
2) Supplementary Methods 1
3) Supplementary Notes 1-3
4) Supplementary Tables 1-7

Supplementary figures

Supplementary figure 1: **Repeat content of the genomes.** Repetitive elements composition in the five assemblies calculated using RepeatMasker, showing the similar compositions of the five genomes.

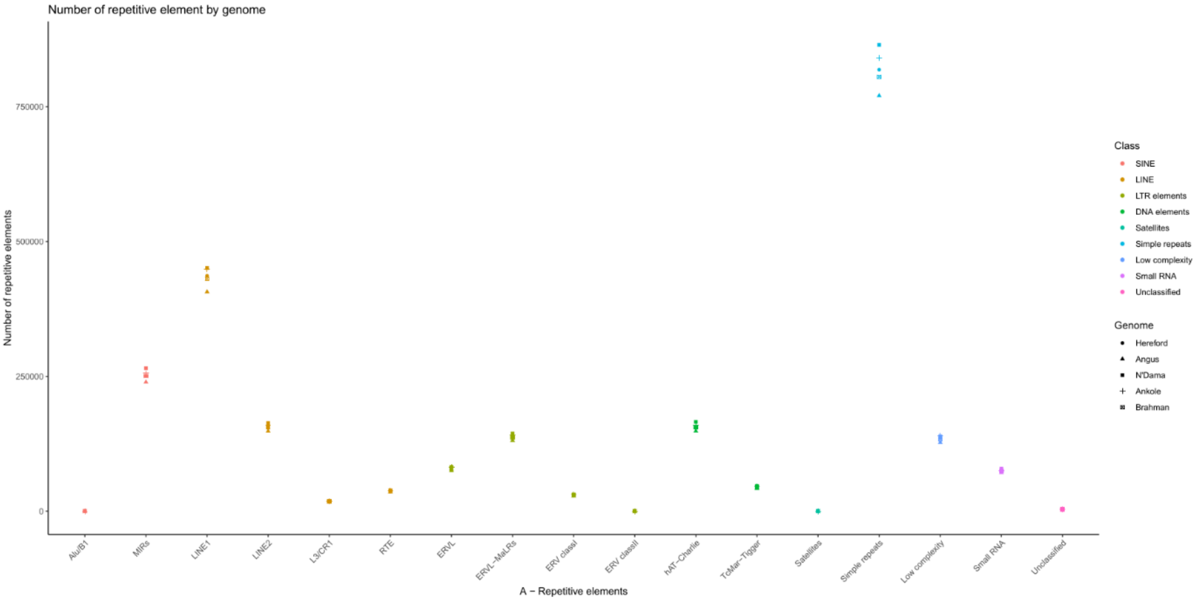

Supplementary figure 2: **MHC diversity.** Alignments generated by minimap2 over the whole chromosome 23, showing the MHC region as a drop in alignment identity in all the assemblies.

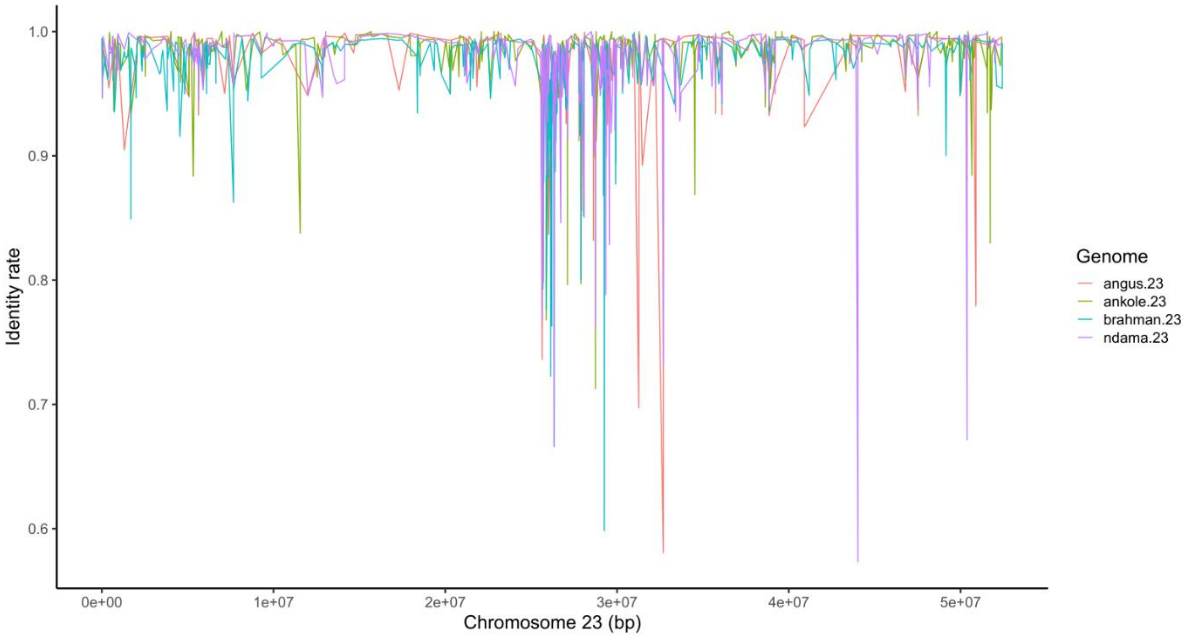

Supplementary figure 3: **Selection on indels in coding regions.** Allele size distribution in intergenic and intragenic portions of the genome, showing how in-frame indels from the graph were more common than other coding indels, consistent with selection disproportionately removing frameshift changes.

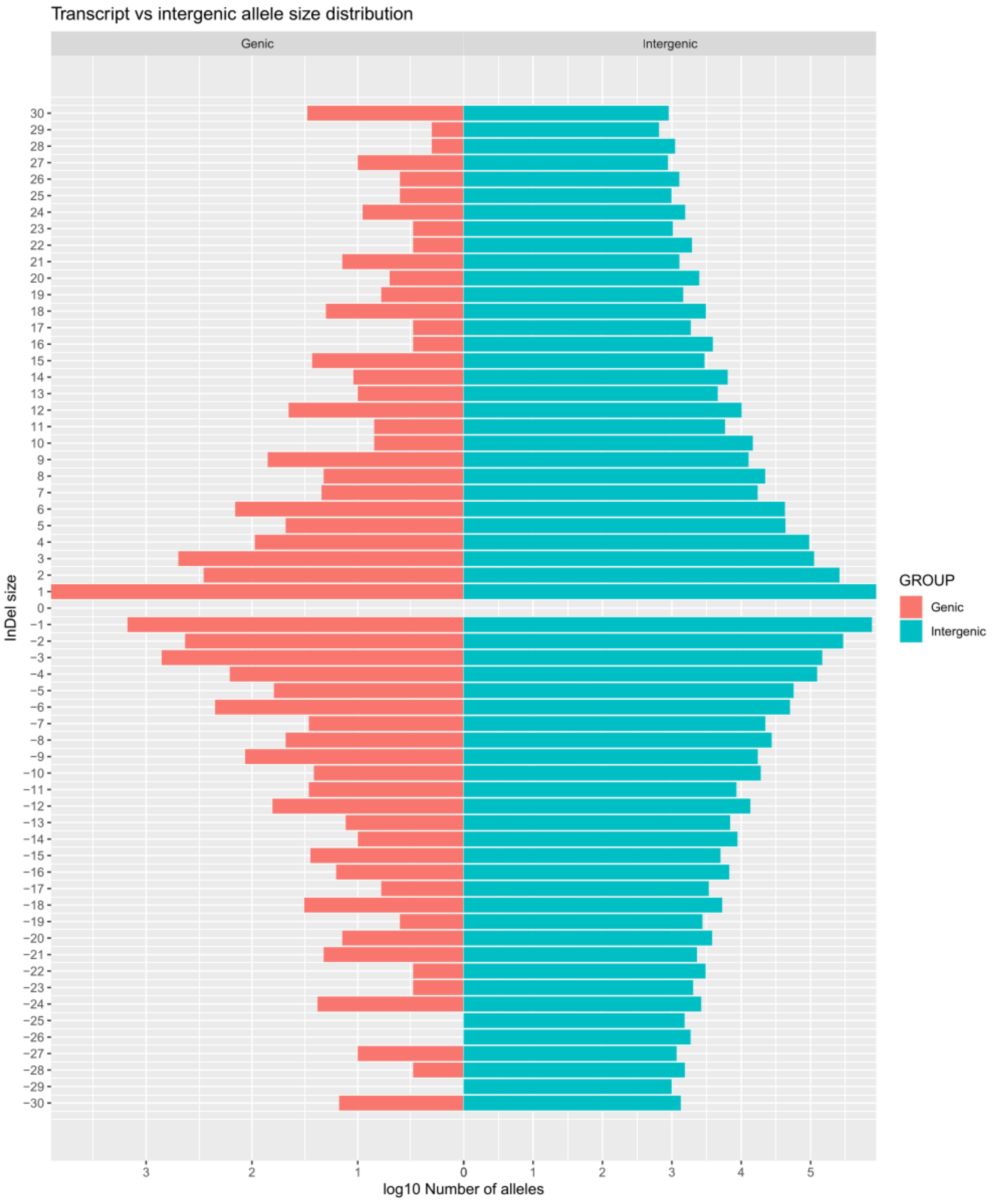

Supplementary figure 4: **Divergence across assemblies on chromosome 12.** Alignment of chromosome 12 of the five assemblies, showing the gap in the N'Dama genome is a high-complexity region across the assemblies.

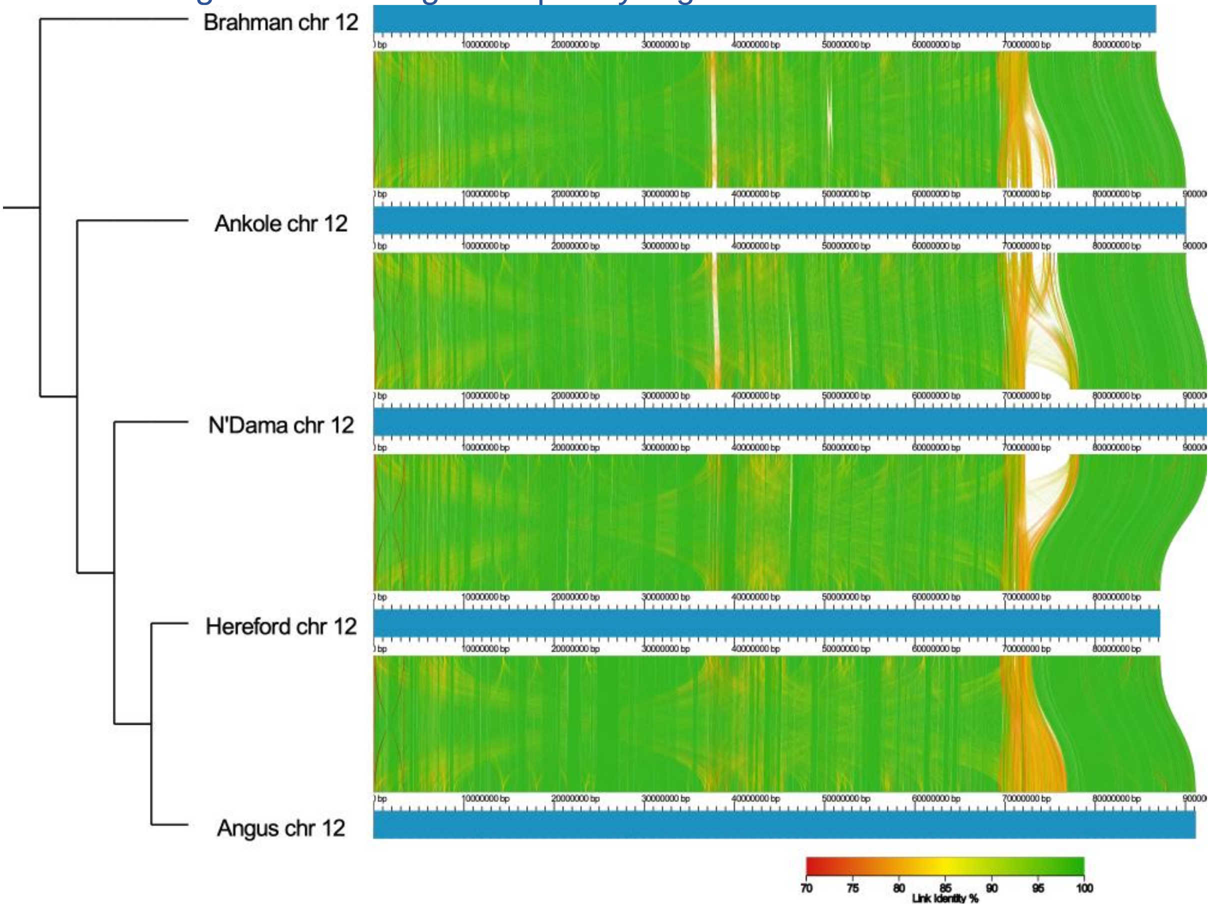

Supplementary methods

Supplementary methods 1 - Detailed description of the preparation of the ATAC-seq samples.

Extraction of PBMC DNA and isolation of B cells

Peripheral blood mononuclear cells (PBMCs) were isolated from Holstein Friesian, N'Dama and Nelore peripheral blood by density gradient centrifugation using Ficoll Plaque Plus (GE Healthcare). DNA was extracted from PBMC using a QIAGEN DNeasy blood and tissue kit with proteinase K and RNase treatment. For isolation of B cells, PBMCs were resuspended at $3x10^7$ cells/ml in PBS/2 mM EDTA/0.5% BSA and incubated with 0.066 µg/ml ILA58 monoclonal antibody, which binds the immunoglobulin light chain, for 20 min at 4°C. After two washes in PBS/2 mM EDTA/0.5% BSA, PBMCs were incubated in 2 ml of 2 µg/ml PE-conjugated goat anti-mouse IgG2a (Molecular probes) for 20 min at 4°C. PBMCs were washed twice and were resuspended in PBS/2 mM EDTA/0.5% BSA with or without 1 µg/ml DAPI (Invitrogen). B cells were sorted on a FACSAria II or III Cell Sorter (BD Biosciences) or BD Influx Cell Sorter (BD Biosciences) with 82 - 97 % purity validated by post-sort flow cytometric analysis of 1,000 events. All cells were stained and sorted within 9 hours of blood collection and kept on ice between processing steps.

ATAC-seq library preparation

Cells of the mouse mastocytoma cell line P815 were spiked into the Holstein Freisian B cell sample at a 1:10 ratio. For the three breeds, 50,000 cells were transferred into a 96-well v-bottomed plate on ice. Cells were centrifuged at 500 x*g*, 4°C for 2 min and the supernatant was removed. Next, cells were resuspended in 100 µl cold lysis buffer (10 mM Tris hydrochloride, pH 7.4, 10 mM sodium chloride, 3 mM magnesium chloride, 0.1% IGEPAL CA-630). Cells were centrifuged at 500 x*g* at 4°C for 10 min and the supernatant was discarded. Nuclei were resuspended in 50 µl transposase mixture (25 µl 2x TD buffer, 2.5 µl TDE1 Tagment DNA (Illumina) and 22.5 µl nuclease-free water), transferred to 1.5 ml microcentrifuge tubes, and then were incubated for 30 min at 37°C in an Eppendorf Thermomixer with agitation at 300 rpm. Transposed DNA was purified using a QIAGEN MinElute Reaction Cleanup Kit with elution in 14 µl water. To generate presumably nucleosome-free ATAC-seq libraries, these steps were repeated using 1500 - 2000 ng Holstein Friesian PBMC DNA treated with protease K in replacement of the 50,000 cells. Transposed DNA was then amplified using Nextera primers listed in Buenrostro et al., 2015[1]. Specifically, all samples were amplified using the index i5 primer v2_Ad1.1_TAGATCGC and one of the index i7 v2_Ad2.1 - 2.12 primers. qPCR reactions were carried out in duplicate using 0.5 µl transposed DNA, 5 µl NEBNext High-Fidelity 2x PCR Master Mix (NEB), 1.25 µl 10 µM dual-index PCR primers (Integrated DNA Technologies), 0.25 µl 20x SYBR Green I (Invitrogen), 0.15 µl 1 mM ROX reference dye (Agilent Technologies), and 1.6 µl nuclease-free water (QIAGEN). The PCR conditions were as follows: 72°C for 5 min, then 98°C for 30 sec, followed by 21 thermocycles at 98°C for 10 sec, 63°C for 30 sec and 72°C for 1 min. To calculate the appropriate number of cycles for amplification of the remaining transposed DNA, linear Rn was plotted against cycle number to determine the cycle number corresponding to one-quarter of the maximum fluorescent intensity, with an average taken across duplicates. This number of cycles was used to amplify the remaining 12.5 µl transposed DNA using 25 µl NEBNext High-Fidelity 2x PCR Master Mix (NEB), 6.25 µl 10 µM dual-index PCR primers, with the same cycling

conditions as described for qPCR. The DNA was purified using a Qiagen MinElute PCR Purification Kit (QIAGEN) with elution in 20 µl nuclease-free water (QIAGEN). To remove residual primers, the libraries were purified using 1.4X AMPure beads (Beckman Coulter). Then, two further AMPure steps were performed to remove large DNA fragments (>1000bp). The first used 0.5X AMPure beads with recovery of the supernatant, to which 1.3X AMPure beads were added to purify the final libraries. Library quality was assessed for the fragment length distribution on a 2200 TapeStation instrument (Agilent Technologies), using High Sensitivity D1000 ScreenTape and Reagents (Agilent Technologies). The library concentrations were measured on a Qubit 3.0 (Invitrogen) using a Qubit dsDNA High Sensitivity assay kit (Invitrogen). Resulting libraries were sequenced using 75 bp paired-end sequencing on a HiSeq 4000 sequencer (Illumina) or 50 bp paired-end sequencing on NovaSeq 6000 sequencer (Illumina) at the Edinburgh genomics facility.

Supplementary Notes

Supplementary Note 1 – In-depth description of the N'Dama assembly process, with detailed metrics and processes

N'Dama long read sequencing

Pre-Assembly statistics of sample's long reads

Alignment statistics to latest Bos taurus assembly via minimap2[2]:

minimap2 -ax map-pb BtauARS.fasta Ndama.subreads.bam | samtools view - -S -h -b | samtools sort - -o ./Ndama.subreads_minimap.bam

Alignment statistics obtained through samtools[3] flagstat.
In total, reads (87.95%) were aligned to the reference genome.

Coverage obtained on the chromosomes (samtools depth) as the average of all bases within each chromosome.

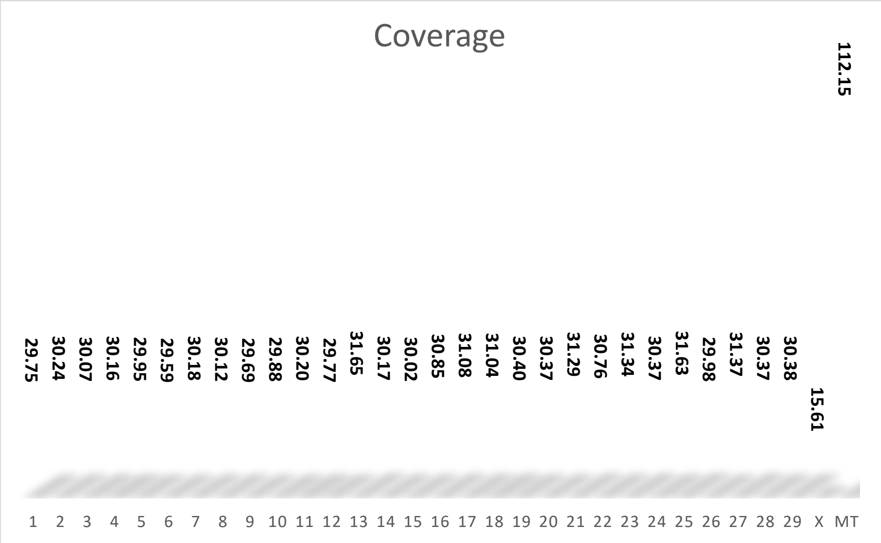

Coverage per chromosome of mapped PacBio reads

Assembly Selection

We generated two different assemblies from two different software, CANU[4] and FALCON[5]. Different metrics used to select the genome for subsequent analysis.

PARAMETER	CANU	FALCON
BP YIELD	2,624,985,022	2,644,771,833
# SEQUENCES (POLISHED)	5,940	4,115
N25	4,717,823	6,209,788
N50	2,636,435	3,275,473
N75	1,195,117	1,561,644
N90	328,738	477,322
N95	99,257	124,586
L25	93	78
L50	285	227
L75	652	518
MIN L	1,007	9,000
AVG L	442,266	641,623
MEDIAN L	44,046	69,448
MAX L	21,377,347	18,329,644
GC%	41.95%	42.04%
BUSCO COMPLETE*	77.90%	85.84%
BUSCO FRAGMENTED*	14.00%	6.99%
BUSCO MISSING*	8.10%	7.16%

Based on the metrics above, FALCON assembly have been selected since it shows a high contig metrics, bp yield and includes a filtering for highly repetitive regions. The resulting assembly has been polished twice using long reads with Racon, and once with short reads and Pilon v1.23[6]. Long reads were mapped using minimap2, whereas short reads were mapped using bwa mem.

Pilon polishing step allowed to fix multiple misassemblies, insertions, deletions, collapse repeated regions and trim low coverage bases. Below a short summary of the polishing step:

	VALUE
**ORIGINAL SIZE**	2,655,094,705
**BASES CONFIRMED**	2,609,970,564
**BASES CONFIRMED (%)**	98.30%
**CORRECTED SIZE**	2,644,876,474
**SNPS CORRECTED**	593,486
**INSERTIONS CORRECTED**	5,129,742
**INSERTIONS CORRECTED (BP)**	8,254,027
**DELETIONS CORRECTED**	566,769
**DELETIONS CORRECTED (BP)**	686,240
**COLLAPSED BASES**	11,721,029

After running Pilon on the assembly created using Falcon-Unzip, we run a completeness assessment using BUSCO[7] v3, with the assembly showing a good completeness (91%).

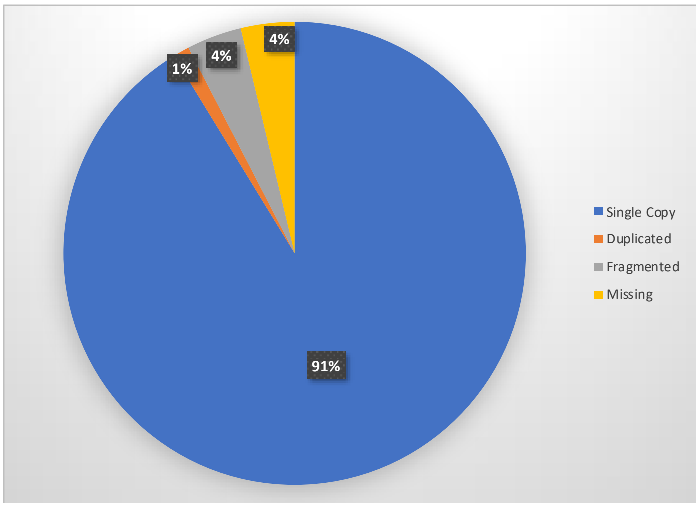

Chromosome-level assembly

Ragout Scaffolding

Lacking long-range sequencing data, we performed the scaffolding of the genome using a reference-assisted approach. Given the availability of multiple reference genomes available, we decided to adopt a multi-reference approach, which might help minimizing the bias introduced by the process. In particular, we decided to use the combination of SibeliaZ[8] v1.1.0 (https://github.com/medvedevgroup/SibeliaZ/) for the alignments and Ragout2[9] (https://github.com/fenderglass/Ragout/) for the scaffolding. We used three different references (ARS-UCD1.2, GCA_003369685.2, GCA_003369695.2, which are respectively Hereford, Angus and Brahman). Sequence names have been changed in the UCSC format (>spp.sequenceID; e.g. >hereford.1), matching the input fasta name.

No phylogenetic tree was provided to the software, leaving to Ragout to estimate the relationships from the alignments. The scaffolding has been performed separately for the autosomes, MT, X, Y and the other contigs and scaffolds. The process is represented in the figure below, with the different steps and the genomes used.

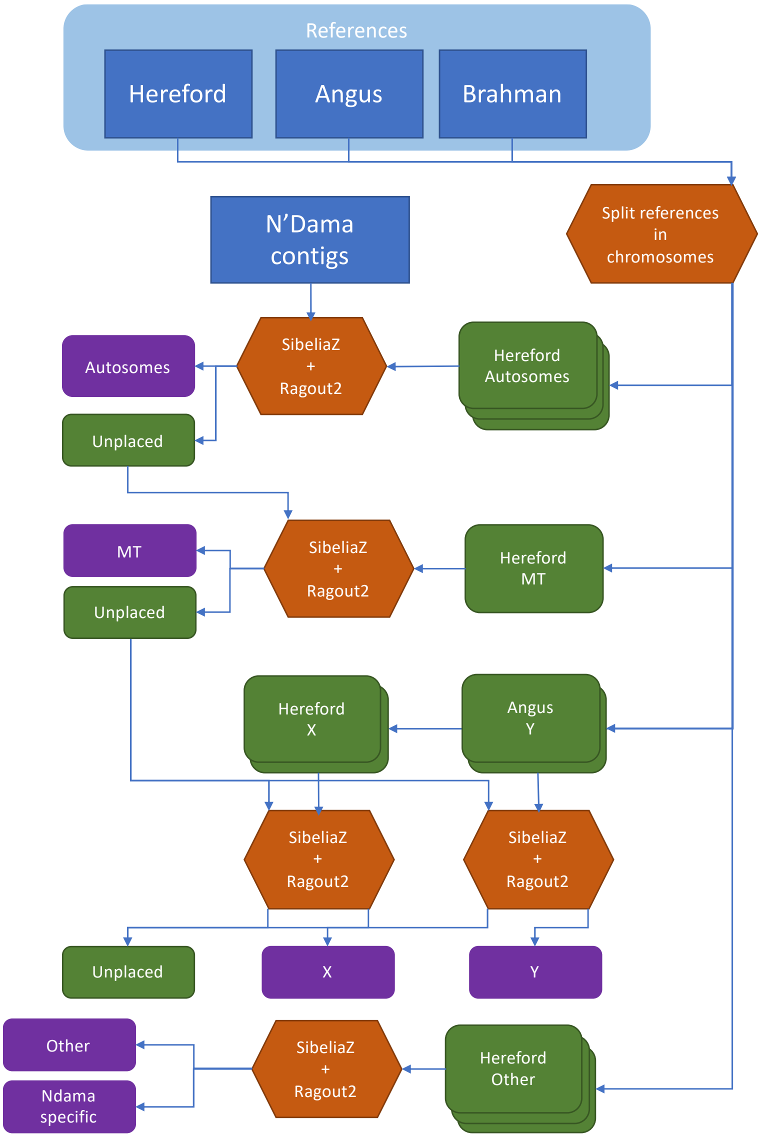

Scheme of the scaffolding step: first, autosomes are scaffolded; the unplaced contigs are first used to create the MT genome, then the remaining used for both X and Y (the same set of contigs have been used to account for pseutoautosomal regions)

SibeliaZ was run with a low k-mer size (-k 21) to use shorter Kmers, slowing down the alignments but increasing the sensitivity.
Most of the contigs are placed on the autosomes, leaving a total sequence length which is roughly the same size as the X chromosome. Worth noticing that one scaffold was identified as on chromosome 7, but the correct position could not be resolved at the time.

BLOCK	FRAGMENT USED	SCFLD LENGTH	INTRODUCED N	INTRODUCED N (%)	UNPLACED FRAGMENTS	UNPLACED LENGTH	UNPLACED LENGTH (%)
AUTOSOME	2,081	2,504,980,383	16,307,546	0.65%	2,564	156,203,637	5.91%
X	2,194	155,751,820	64,638,795	41.50%	1,342	65,056,028	41.66%
Y	640	47,396,136	8,747,893	18.46	2,157	117,520,810	75.25%
MT	1	34,584	0	0.00	2,563	156,169,053	99.98%
OTHER	0	0	0	0.00	1,177	62,647,416	100.00%

The sexual chromosomes present a particularly high number of missing bases, due to the lower coverage derived from the sequencing. Also, the mitogenome, presents twice the expected size. These were considered separately and fixed manually by orientating the fragments and including a gap of ~400 bp.

Mitochondria misassembly resolution

Approach description

Using the QUAST[10] report on the error corrected contigs, it is possible to identify the region of misassembly into the mitochondrial genome.

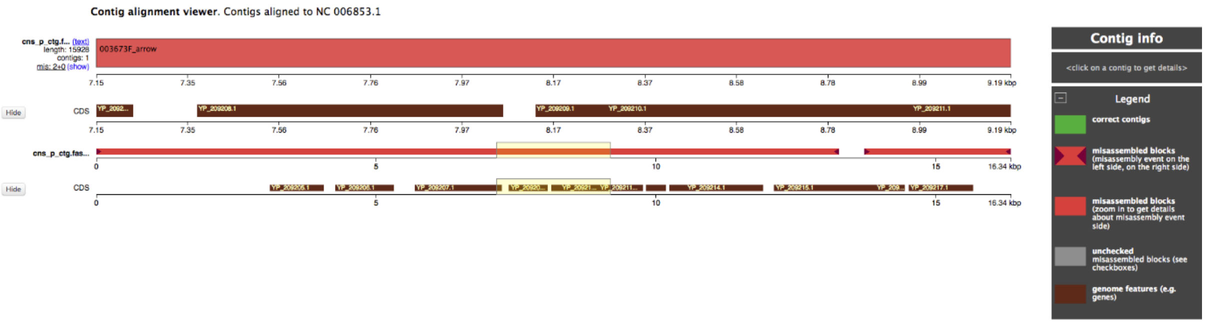

To solve the misassembly, assembled mitochondrial chromosome was aligned to the reference mitochondrial genome using minimap2. Paf alignments were converted to MAF (multiple alignment format). Sorted alignments were then joined manually including a 454 bp gap, reaching the final chromosomal genome length.

Scaffold N/L statistics

We calculated some basic statistics of the new chromosome level assembly (Table below). As can be seen, the N50/L50 are high, as expected from a chromosome-level genome, and 90% of the total genome is included in the chromosomes.

X	NX	LX	NGX	LGX	GC%
5	142,323,786	0	142,323,786	0	40.30
10	126,884,692	1	121,213,004	2	40.56
25	103,955,525	5	103,955,525	5	41.08
50	87,672,696	12	87,672,696	12	40.97
75	61,733,321	21	61,151,627	22	41.60
90	36,413,080	29	9,265,313	32	41.85
95	4,616,499	45	2,179,503	62	41.90
100	7,612	970	7,612	970	42.00

Gap Filling

We improved the contiguity of the assembly through gap filling using the software LR_GapCloser[11] v1.1. This software split the long reads into 300 bp chunks and map them to the scaffolds using bwa. After that step, it identifies the reads than cover mostly the gap, and use them to fill it. The gap fill stage has been repeated three times. As can be seen in table below, ~50% of the gaps and 34% of Ns have been properly filled:

STAGE	N GAPS	GAPS BP	BASES	ADDED BP	N50	L50
INITIAL	4,885	89,694,751	2,769,745,704	0	2,868,616	260
ITERATION1	2,654	63,679,091	2,769,864,878	119,174	10,018,179	77
ITERATION2	2,508	60,276,639	2,769,873,814	8,936	10,310,270	75
ITERATION3	2,480	59,007,146	2,769,874,172	358	10414461	74

Genome polishing

Following the gap filling, the genome needs to be finalized through a 5-fold polishing iteration using the short reads and Pilon (v1.23). Illumina short reads (coverage 78X) have been aligned using bwa mem algorithm in 18 chunks, combined with bamtools[12] 2.4.2 and sorted with samtools 1.9.

Below, a table summarising the changes introduced by multiple Pilon runs. Each iteration involved the remapping of the reads to the latest polished version of the assembly.

PILON RUN	TOT CHANGES	INDELS (<=5BP)	INDELS (>5BP)	#SNP	#GAPS FIXED
**1**	1,967,205	1,305,821	75,113	586,062	209
**2**	268,386	107,525	37,079	123,774	8
**3**	97,875	32,851	23,049	41,973	2
**4**	54,323	12,559	19,041	22,719	4
**5**	38,538	6,470	17,214	14,852	2

Every iteration reduces the number of changes needed by the assembly. The second Pilon run changed 25% SNPs changed in run 1 (120K vs 580K). The third run changed 33% of SNPs changed from run2 (42K vs 124K), and run 4 changed ~50% of SNPs changed in run3 (22K vs 42K). Similar pattern, but stronger, can be observed with the large indels, that at every iteration decrease massively (from >1,300K to ~12.5K from run 1 to 4). Short indels are less present, and therefore their reduction is less outstanding. However, since small insertions/deletions are a known issue in PacBio sequencing, having a low number of these events confirm that the polishing is proceeding in the right direction. Finally, the gaps fixed changes at every iteration. With the exception of the first, that fixed >200 small gaps, the number of gaps fixed in subsequent iterations depends on how much sequence is added in the previous step. Therefore, it can happen that iteration 4 fix more gaps than iteration 3 (4 vs 2).

Genome evaluation

Contigs and scaffold metrics for the final assembled genome have been calculated using an in-house script. The quality value (QV) have been calculated using merqury[13] (https://github.com/marbl/merqury) on the k-mer counts generated with meryl 1.2 (https://github.com/marbl/meryl). QUAST-LG v5[10] (http://quast.sourceforge.net) and FRC_Align (https://github.com/vezzi/FRC_align) have been run to assess the genome using a separate reference and an independent evaluation through short reads sequencing. Coverage of the non-N sequence have been calculated with samtools.

	N'DAMA
SCAFFOLDS N50	104,847,410
SCAFFOLDS L50	11
CONTIGS N50	10,726,776
CONTIGS L50	72
GAPS	2,425
QUAST GENOME FRACT.	93.9
QUAST MISASSEMBLIES	7,050
AUTOSOMAL GAPS	792
QV	34.3
QV (AUTOSOMES)	37.9

The final busco assembly shows 94% of completeness, of which 92.6% in single copy.

BUSCO	N	%GE
COMPLETE	3860	94.1%
COMPLETE (S)	3801	92.6%
COMPLETE (D)	59	1.4%
FRAGMENTED	124	3.0%
MISSING	120	2.9%
TOTAL	4104	100.0%

Coverage plots for the assembly showing the highest level of coverage around the value of 84X (expected = 80X).

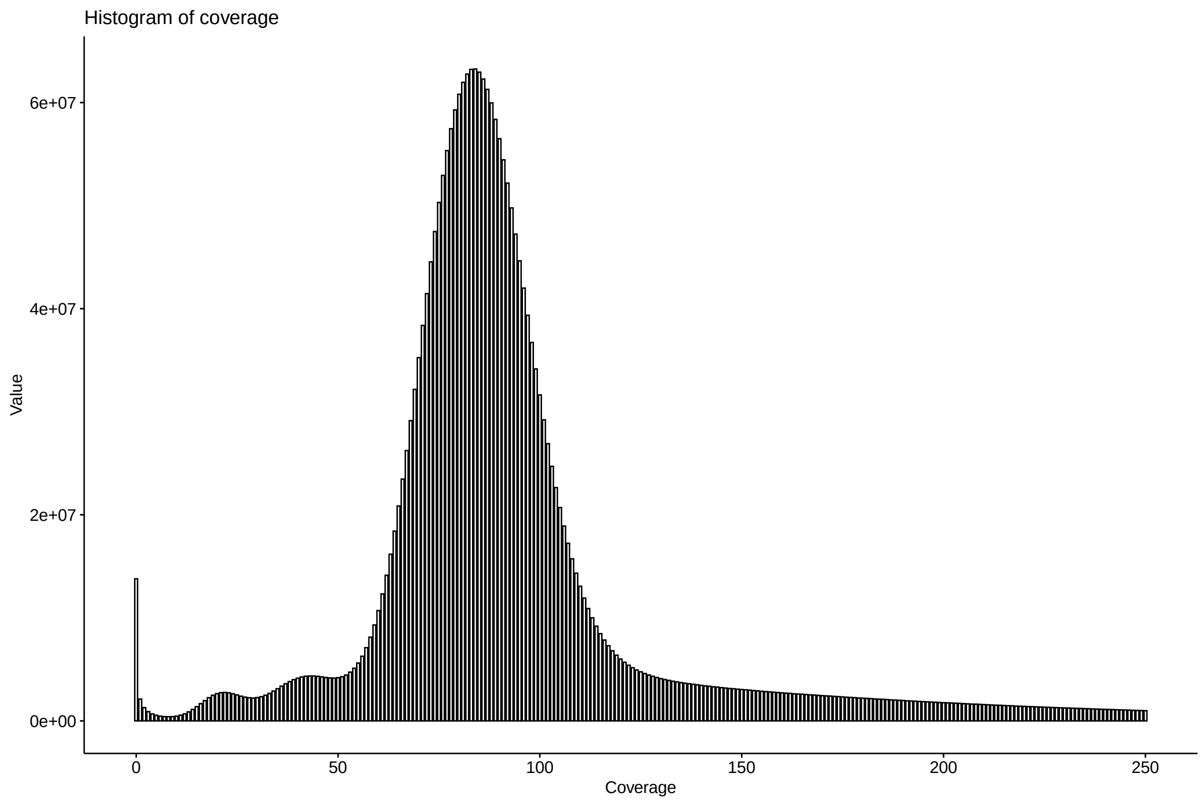

Feature response curve generate through FRC_Align for the assembly is reported below.

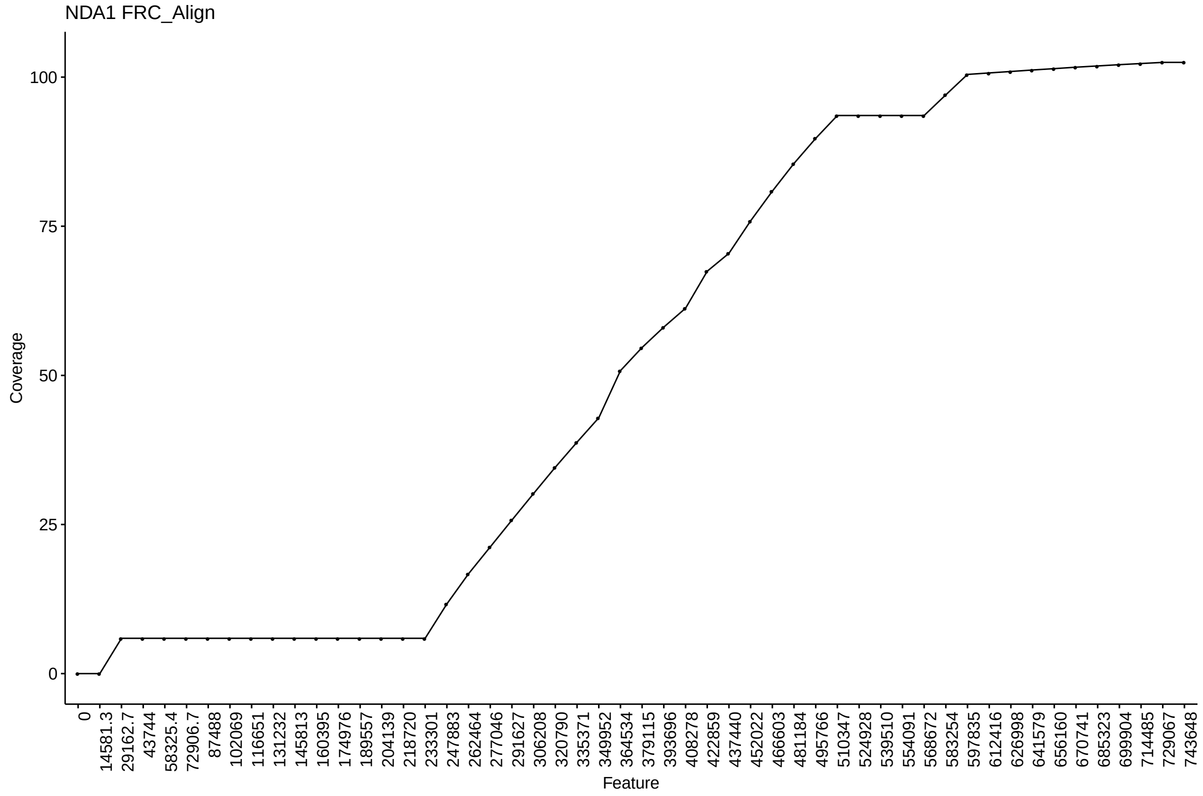

Repetitive region detection and masking

Following the generation of the genome we performed the masking of the repetitive elements. To identify and mask the repetitive regions, we use a combination of:

1. Dustmasker from NCBI blast+[14] tool, to mask low-complexity repetitions
2. Windowmasker, to mask interspersed repeats
3. RepeatMasker[15], that mask interspersed repeats, but that also include trf to mask low-complexity regions

This software generate a bed file with the position of all the repetitive elements in the genome that are then masked using bedtools[16] maskfasta function.

The run of the tools is scattered over multiple jobs, each processing several contigs, to speed up the process. Results from RepeatMasker are then summarised using in-house python script.

Code availability

All scripts used to generate the assembly are available on GitHub https://github.com/evotools/CattleGraphGenomePaper/tree/master/Assembly/NDA1.

Supplementary Note 2 – In-depth description of the Ankole assembly process, with detailed metrics and processes

Ankole long read sequencing

Raw Sequel reads metrics

In table below, there are the metrics for the raw PacBio Sequel subreads.

Parameter	Value
#Reads	9,781,220
Sum	103,164,533,592
Coverage	38.21
Minimum	50
Maximum	134,087
Mean	10,547.21
Standard dev.	8,851.69
Median	8,448
IQR	12,813

Table 1 – Raw reads base statistics

These parameters confirm the general coverage (~40X) and that the reads seems to have a good median length.
Below, Figure 1 shows the read lengths distribution:

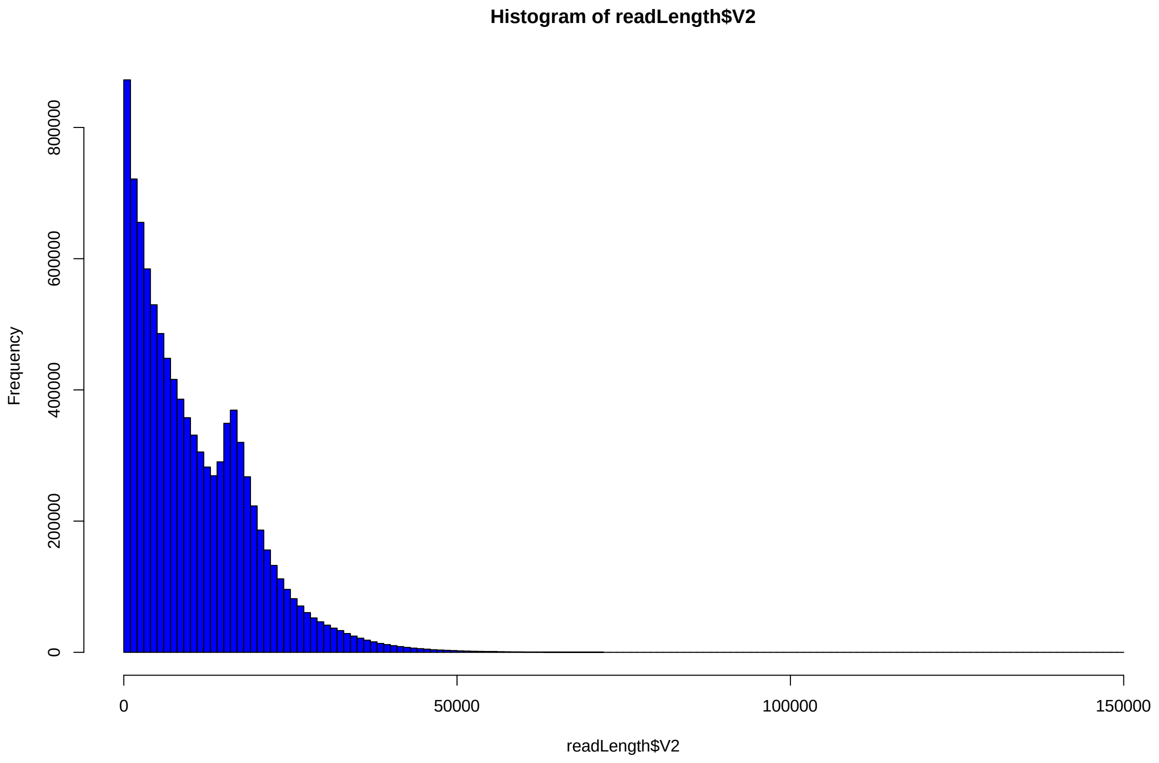

Figure 1 – Read lengths distribution

Analogously to the N'Dama assembly, the reads shows a bimodal distribution, with a first peak to low lengths (<1Kb) and a second at ~17Kb.

Assembly

We generated two different assemblies using two different assemblers: CANU and WTDBG2. The scripts used to generate the two genomes are reported in GitHub repository. Both assemblies have been polished through WTPOA-cns tool included with the WTDBG2[17] software. The table below shows the differences between the two assembled genomes.

PARAMETER	WTDBG2	CANU
N50	2,325,045	1,800,361
L50	329	441
BP yielded	2,703,598,714	2,807,643,510
# Sequences	10,809	10,545
BUSCO Complete*	89.6%	84.5%
BUSCO Fragmented*		
BUSCO Missing*		

Assembler comparison

Both CANU and WTDBG2 provided similar results, with contigs N50 above 1Mb with WTDBG2 showing a slightly higher N50 and lowest L50. Despite that, CANU[4] generated less contigs (300 contigs less) and a longer assembly size (2.8Gb vs 2.7Gb). Most of the statistics still need to be computed for CANU and FALCON, since the two assembler are respectively running the polishing step and generating the contigs.

Assembly reconciliation tools

Following assembling the genomes, we tried to improve the contiguity of the genome by generating applying a genome reconciliation tool. We used quickmerge[18], a software that relies on MUMmer[19] alignments filtered for repetitive regions, to detect the overlaps among contigs and combine them into new, longer sequences.

The genomes have been aligned using the nucmer tool in MUMmer4 suite, filtering all the repetitive regions.

The resulting alignments are then used as input for quickmerge, that has been run allowing length cutoff longer than the highest N50 (2.5Mb), considering a minimum alignment length of 50Kb (10X higher than the recommended threshold) and with very stringent cutoffs for the alignments considered for merging (hco > 15, 3X higher than the recommended; c > 5, 3.3X higher than the recommended; values are analogous to [20]).

	Value
**BP yielded**	2,808,308,196
**N50**	8,161,114
**L50**	94
**N sequences**	9,270
**GC%**	41.99

The procedure might have introduced misassemblies, or consolidated some that were present in both assemblies. The use of the Bionano optical mapping will likely fix the largest of these, breaking up chimeric contigs into smaller fragments.

BioNano optical mapping scaffolding

Software description

The generation of scaffolds have been performed using the optical mapping (OM) generated with the BioNano Saphyr machine. Molecules generated have been processed through the BioNano Solve 3.3 pipeline (version 7981).
The optical maps have first been assembled using the quickmerged assembly as reference, and then scaffolded using the hybrid scaffolding pipeline. The workflow introduced 956 cuts to 65 NGS sequences out of 9,271 (0.7% of the total; see table below for output from the conflict solution).

	VALUE
**NUMBER OF CONFLICT CUTS MADE TO BIONANO MAPS**	170
**NUMBER OF CONFLICT CUTS MADE TO NGS SEQUENCES**	956
**NUMBER OF BIONANO MAPS TO BE CUT**	165
**NUMBER OF NGS SEQUENCES TO BE CUT**	65

Eighteen out of the 65 contigs that need to be cut were above 2.5Mb, thirteen between 1Mb and 2.5Mb and 31 below 1Mb, with all contigs that had more than 5 cuts below the 1Mb threshold, suggesting the presence of misassemblies at the assembler level. More than 60% of the contigs (40/65) were split in two fragments, 24% in three fragments and the 15% in >4 fragments.

CTG ID	CTG SIZE	# FRAGMENTS	SMALLER FRAGMENT SIZE	LARGER FRAGMENT SIZE	ALL FRAGMENT SIZES
**6399**	235,114	10	2608	120196	41052;7461;13523;12100;4012;2608;5415;26130;2608;120196
**3144**	163,846	9	2559	115541	28708;2559;4002;2604;2608;2605;2605;2606;115541
**2772**	371,428	7	59	158049	50584;40337;59;80208;17888;24297;158049
**3438**	113,603	7	3989	29329	27292;3989;7749;18511;22719;4008;29329
**637**	794,184	5	59	398055	163493;4325;228248;59;398055
**1731**	887,286	5	21386	443612	82670;79794;443612;21386;259820
**1915**	811,417	4	10574	559832	559832;10574;10846;230162
**9129**	12,039,443	4	59	8774036	3237364;27981;59;8774036
**9247**	12,474,065	4	8208	12092034	12092034;48131;8208;325689
**1147**	1,585,356	3	23507	1206018	355829;23507;1206018
**1270**	1,331,647	3	6379	732968	732968;6379;592298
**2109**	322,094	3	22980	245702	53410;22980;245702
**2157**	618,540	3	13853	568760	35925;13853;568760
**2322**	453,240	3	9931	302333	302333;9931;140974
**3383**	109,369	3	2608	76059	30700;2608;76059
**3428**	142,634	3	4012	94822	43798;4012;94822
**5960**	2,404,879	3	6436	2001399	2001399;6436;397042
**6007**	609,323	3	39787	490829	78705;39787;490829
**6237**	256,704	3	10727	161749	161749;10727;84226
**6413**	88,473	3	4419	58615	58615;4419;25437
**8727**	1,889,629	3	23394	1472041	394192;23394;1472041
**9094**	50,349,372	3	6311	28628641	21714418;6311;28628641
**9143**	21,002,380	3	32861	13946044	13946044;32861;7023473
**9194**	2,812,833	3	28719	2686466	97646;28719;2686466
**9229**	8,027,832	3	59	4443568	3584203;59;4443568
**8**	4,496,967	2	1853501	2643465	1853501;2643465
**72**	181,619	2	32189	149429	32189;149429

**216**	182,234	2	46042	136191	46042;136191
**594**	2,496,872	2	29398	2467473	29398;2467473
**629**	1,263,114	2	23285	1239828	1239828;23285
**800**	1,114,663	2	445969	668693	445969;668693
**972**	1,878,577	2	235915	1642661	235915;1642661
**1084**	1,568,556	2	730844	837711	730844;837711
**1312**	1,409,293	2	104420	1304872	104420;1304872
**1412**	1,204,556	2	181058	1023497	1023497;181058
**1868**	876,811	2	106805	770005	770005;106805
**2130**	445,190	2	119237	325952	119237;325952
**2447**	381,485	2	90006	291478	291478;90006
**2809**	122,592	2	36774	85817	85817;36774
**2968**	122,937	2	43020	79916	79916;43020
**3311**	141,503	2	32150	109352	32150;109352
**5993**	1,182,297	2	64329	1117967	64329;1117967
**6037**	2,348,223	2	133913	2214309	2214309;133913
**6058**	245,737	2	60375	185361	60375;185361
**6143**	288,439	2	135856	152582	152582;135856
**6168**	508,293	2	51190	457102	457102;51190
**6230**	270,168	2	75577	194590	194590;75577
**6251**	210,208	2	98570	111637	98570;111637
**6253**	332,684	2	37785	294898	37785;294898
**6256**	405,643	2	36263	369379	36263;369379
**6360**	357,635	2	117202	240432	240432;117202
**9103**	13,274,084	2	54086	13219997	13219997;54086
**9126**	5,989,548	2	391061	5598486	391061;5598486
**9133**	5,315,080	2	136169	5178910	136169;5178910
**9167**	13,510,537	2	158305	13352231	158305;13352231
**9182**	5,134,164	2	1835694	3298469	3298469;1835694
**9189**	10,293,330	2	1722992	8570337	8570337;1722992
**9193**	6,841,681	2	102276	6739404	102276;6739404
**9204**	8,229,963	2	2445330	5784632	2445330;5784632
**9206**	4,142,798	2	1118188	3024609	3024609;1118188
**9211**	6,756,289	2	1091867	5664421	5664421;1091867
**9219**	9,152,709	2	742149	8410559	8410559;742149
**9241**	16,694,204	2	1420161	15274042	1420161;15274042
**9252**	8,673,619	2	254878	8418740	254878;8418740
**9265**	11,484,869	2	74937	11409931	74937;11409931

The resulting scaffolds, in comparison with the contig level assemblies, show the following metrics:

Assembly	Bp	#Scf	scfN50 (Mb)	#Ctg	ctgN50	ctgL50	#Ns	#Gaps	LongestGap
WTDBG2	2,703,598,714	NA	NA	10,809	2,325,045	329	NA	NA	NA
CANU	2,807,643,510	NA	NA	10,545	1,800,361	441	NA	NA	NA
QUICKMERGE	2,808,308,196	NA	NA	9,270	8,161,114	94	NA	NA	NA
QKM_BNSCF	2,808,308,196	7,581	85.414	9,388	7,823,042	97	118,404,067	1,807	5,536,000

The next stage will be the gap filling and polishing of the genome generated from the Solve pipeline.

Gap Filling

Following the definition of the scaffolded version of the genome, the next step to be performed is the gap filling in order to increase the contiguity of the assembly. We used the scaffolds generated starting from the quickmerge contigs and the raw PacBio long reads. The software used to perform this step is LR_GapCloser[11]. The following table shows the results of each of the 3-fold gap-filling iterations, with the sequential improvements:

Assembly	*Bp*	*#Scf*	*scfN50*	*#Ctg*	*ctgN50*	*ctgL50*	*#Ns*	*#Gaps*	*LongestGap*
WTDBG2	2,703,598,714	NA	NA	10,809	2,325,045	329	NA	NA	NA
CANU	2,807,643,510	NA	NA	10,545	1,800,361	441	NA	NA	NA
QUICKMERGE	2,808,308,196	NA	NA	9,270	8,161,114	94	NA	NA	NA
QKM_BNSCF	2,808,417,765	7,581		9,388	7,823,042	97	118,407,067	1,807	5,536,000
QKM_BNSCF LRGC (I1)	2,816,250,667	7,581		8,822	7,823,042	52	98,366,957	1,241	5,508,511
QKM_BNSCF LRGC (I2)	2,817,716,955	7,581		8,575	16,946,366	49	90,935,046	994	5,508,511
QKM_BNSCF LRGC (I3)	2,818,214,162	7,581		8,505	17,812,669	49	86,562,567	924	5,508,511

Next step in this analysis involves the polishing of the scaffolds, using Illumina short-reads, to further improve the overall quality of the genome.

Genome polishing

Following the gap filling, the genome was finalized through 5-fold iterations of polishing through Pilon v1.23 with 78X Illumina short reads mapped scaffolded, gap-filled genome. Illumina short reads have then been aligned through bwa mem algorithm in 18 chunks, joined with bamtools[12] 2.4.2 and sorted with samtools[3] 1.9.
Below, a table summarising the changes introduced by multiple Pilon[6] runs. Each iteration involved the remapping of the reads to the latest polished version of the assembly.

PILON RUN	TOT CHANGES	INDELS (<=5BP)	INDELS (>5BP)	#SNP	#GAPS FIXED
**1**	4,099,358	3,385,056	135,069	579,233	7
**2**	428,490	207,288	66,250	154,952	8
**3**	155,155	72,104	31,409	51,642	4
**4**	75,285	23,964	26,429	24,892	1
**5**	50,588	12,951	23,749	13,888	1

Every iteration reduces the number of changes needed by the assembly. The number of gaps fixed in each iteration varies depending on the added sequence from the previous iteration.

Chromosome assignment

Alignments and assignments

Following completion of the assembly, we tried to identify which scaffolds corresponded to the autosomes, sexual chromosomes and mitogenome. To do so, we first aligned the scaffolds to the 1000 bull reference genome using minimap2. The resulting paf were then processed through a custom R scripts to extract the alignments that better suited each autosome. The result is reported in the table below, with most of the chromosomes corresponding to a single scaffold, with high percentage of identity:

**Query**	**Target**	**Aligned**	**Tgt Length**	**Qry Length**	**Ratio Qry aligned**	**Ratio Tgt aligned**
Super-Scaffold_100001	1	155901980	158534110	156527526	0.9833971	0.9960036
Super-Scaffold_100002	2	137697348	136231102	138302011	1.0107629	0.995628
Super-Scaffold_100003	3	119614633	121005158	120813661	0.9885085	0.9900754
Super-Scaffold_100005	4	118799771	120000601	119914157	0.9899931	0.9907068
Super-Scaffold_100004	5	116688774	120089316	122124786	0.9716832	0.9554881
Super-Scaffold_100007	6	111516922	117806340	112516630	0.9466122	0.991115
Super-Scaffold_100008	7	109482276	110682743	110533585	0.989154	0.9904888
Super-Scaffold_100006	8	112418051	113319770	114147848	0.9920427	0.984846
Super-Scaffold_100010	9	104081408	105454467	104606559	0.9869796	0.9949798
Super-Scaffold_100011	10	100737099	103308737	103296156	0.9751073	0.975226
Super-Scaffold_100009	11	105979159	106982474	107065538	0.9906217	0.9898531
Super-Scaffold_100012	12	83734362	87216183	89974165	0.9600783	0.9306489
Super-Scaffold_9109	13	82263160	83472345	83163653	0.9855139	0.989172
Super-Scaffold_9214	14	79091931	82403003	82216954	0.9598186	0.9619905
Super-Scaffold_100013	15	81771760	85007780	84415766	0.9619327	0.9686788
Super-Scaffold_100016	16	73121424	81013979	74962467	0.9025779	0.9754405
Super-Scaffold_100017	17	72180629	73167244	72786150	0.9865156	0.9916808
Super-Scaffold_9192	18	65305312	65820629	67291221	0.9921709	0.9704878
Super-Scaffold_100021	19	62919788	63449741	63302509	0.9916477	0.9939541
Super-Scaffold_100018	20	71188688	71974595	72406059	0.9890808	0.9831869
Super-Scaffold_100019	21	68731579	69862954	70766496	0.9838058	0.9712446
Super-Scaffold_100023	22	59638881	60773035	61182121	0.9813379	0.9747763
Super-Scaffold_100024	23	50754560	52498615	52932239	0.966779	0.9588591
Super-Scaffold_100022	24	62068383	62317253	62232639	0.9960064	0.9973606
Super-Scaffold_100030	25	42510937	42350435	42999529	1.0037899	0.9886373
Super-Scaffold_100025	26	50788676	51992305	51362451	0.9768499	0.9888289
Super-Scaffold_100028	27	43889740	45612108	45509546	0.9622388	0.9644073
Super-Scaffold_100029	28	37006736	45940150	42633662	0.8055423	0.8680168
Super-Scaffold_100026	29	51374455	51098607	53282723	1.0053983	0.964186
tig00008859_obj	MT	35866	16340	36534	2.1949816	0.9817157
tig00009004_obj	MT	16102	16340	17364	0.9854345	0.9273209
tig00009055_obj	MT	16988	16340	17519	1.0396573	0.9696901

tig00009122_obj	MT	14966	16340	16578	0.9159119	0.9027627
tig00009170_obj	MT	15444	16340	17012	0.9451652	0.9078298
tig00009193_obj	MT	13312	16340	16003	0.8146879	0.831844
tig00009207_obj	MT	13443	16340	14566	0.822705	0.9229027
tig00009254_obj	MT	14195	16340	15426	0.8687271	0.9201997

The lower identity of chromosome 28 can be linked to the presence of a large gap (>5Mb) reducing the total alignments percentage. Mitogenome has been assembled multiple times in several contigs. The one closer in size to the actual mitogenome is *tig00009055_obj*, which we suggest as the actual mitochondrial genome. Finally, both X and Y have not been properly scaffolded. They are covered by a various number of scaffolds and/or contigs, and never reaching the total expected chromosomal length.

Genome evaluation

Contigs and scaffold metrics for the final assembled genome have been calculated using an in-house script. The quality value (QV) have been calculated using merqury[13] (https://github.com/marbl/merqury) on the k-mer counts generated with meryl 1.2 (https://github.com/marbl/meryl). QUAST-LG v5[10] (http://quast.sourceforge.net) and FRC_Align (https://github.com/vezzi/FRC_align) have been run to assess the genome using a separate reference and an independent evaluation through short reads sequencing. Coverage of the non-N sequence have been calculated with samtools.

	**ANKOLE**
**SCAFFOLDS N50**	84,415,766
**SCAFFOLDS L50**	12
**CONTIGS N50**	18,610,934
**CONTIGS L50**	49
**GAPS**	904
**AUTOSOMAL GAPS**	296
**QUAST GENOME FRACT.**	94.0%
**QUAST MISASSEMBLIES**	5,907
**QV**	30.6
**QV (AUTOSOMES)**	34.2

The final busco assembly shows 93% of completeness, of which 91.0% in single copy.

BUSCO	N	%GE
**COMPLETE**	3819	93.1%
**COMPLETE (SINGLE)**	3733	91.0%
**COMPLETE (DUPLICATE)**	86	2.1%
**FRAGMENTED**	125	3.0%
**MISSING**	160	3.9%
**TOTAL**	4104	100.0%

The coverage plot shows the highest coverage at 89X.

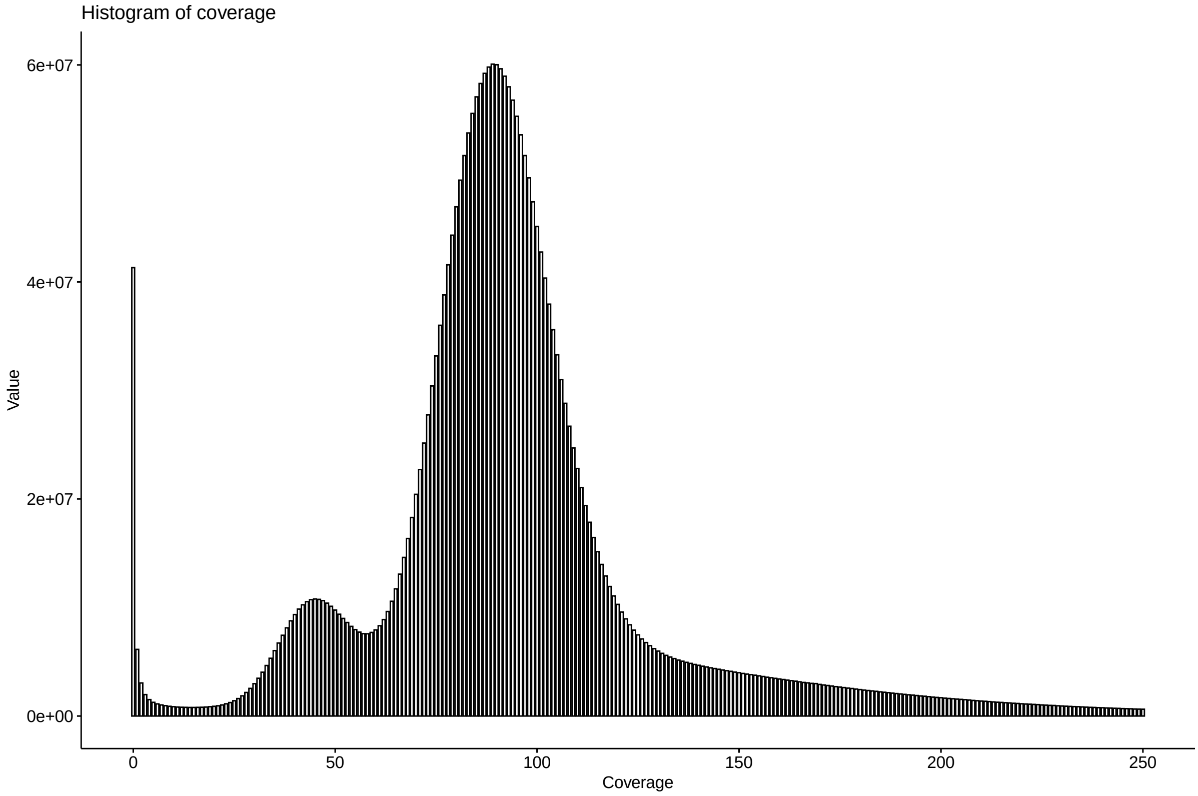

The unusual increase in lower coverage is due to the sparse unplaced contigs, and when removed the second peak almost disappear:

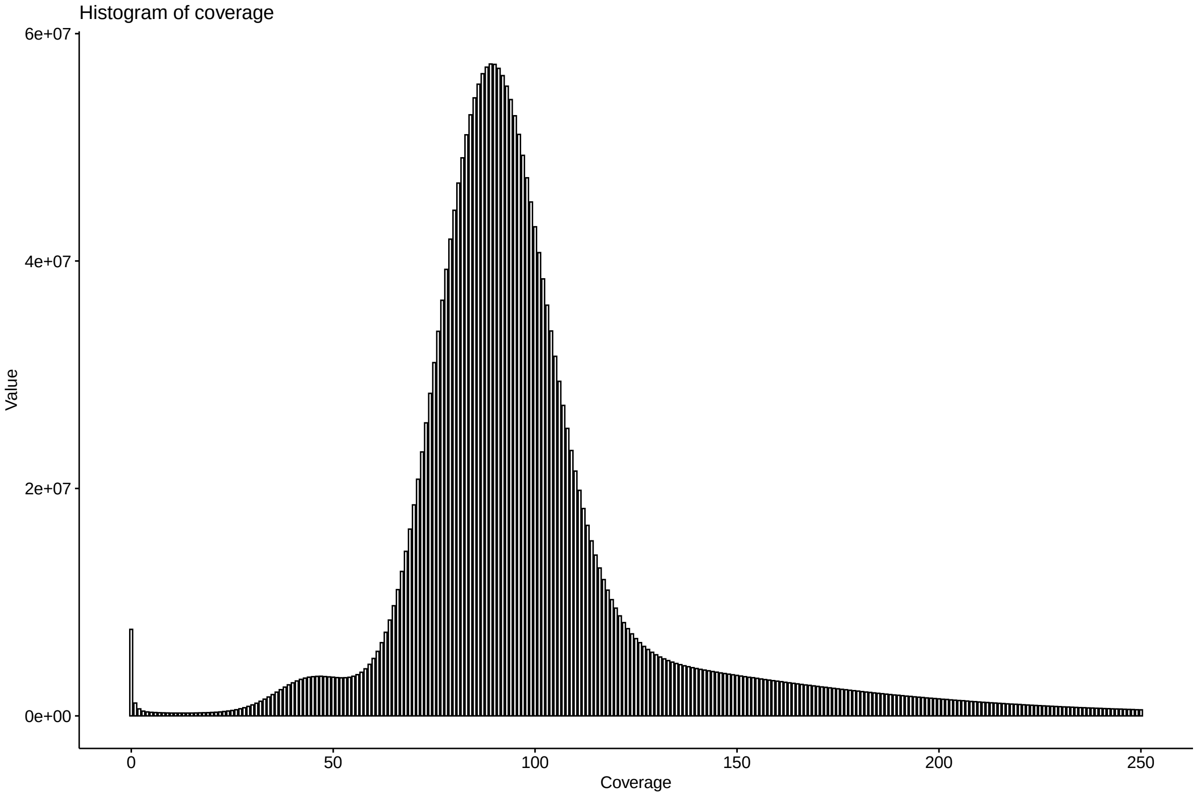

The Feature Response Curve generated with FRC_Align is reported below.

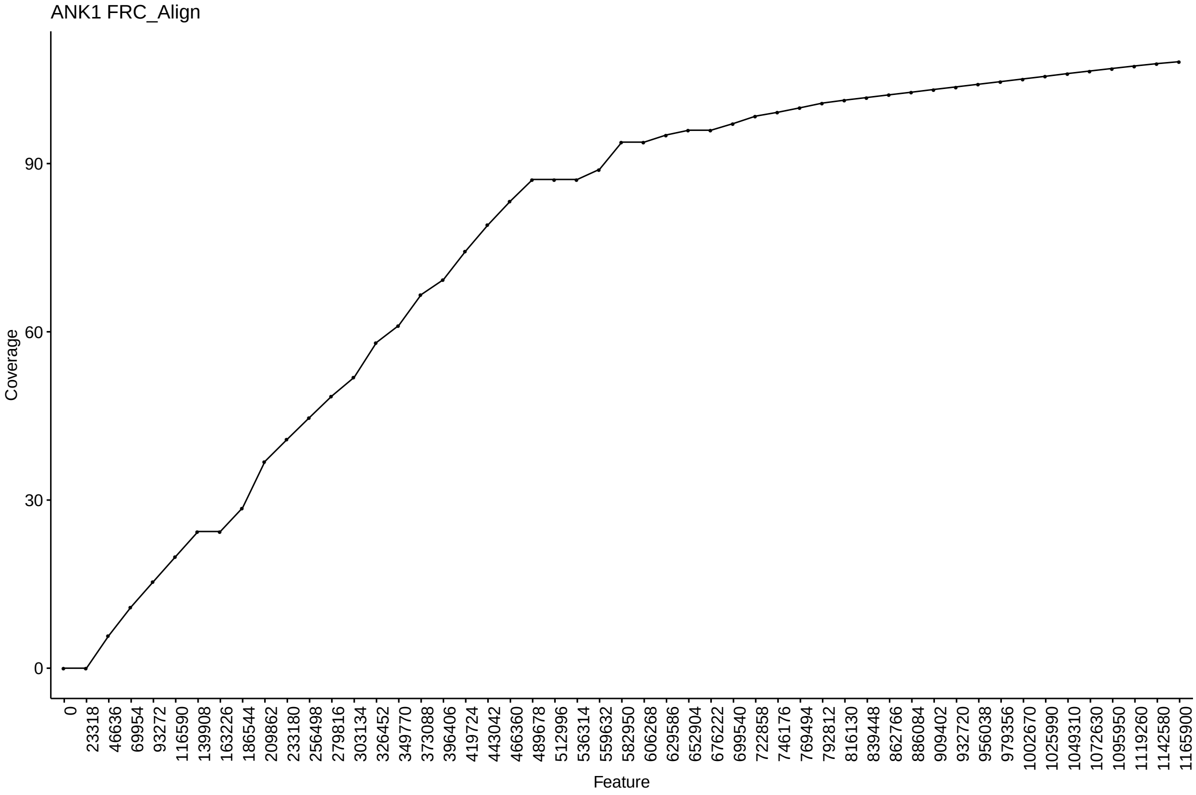

Repetitive region detection and masking

Following the generation of the genome we performed the masking of the repetitive elements. To identify and mask the repetitive regions, we use a combination of:

1. Dustmasker from NCBI blast+ tool, to mask low-complexity repetitions
2. Windowmasker, to mask interspersed repeats
3. RepeatMasker[15], that mask interspersed repeats, but that also include trf to mask low-complexity regions

This software generate a bed file with the position of all the repetitive elements in the genome that are then masked using bedtools[16] maskfasta function.

The run of the tools is scattered over multiple jobs, each processing several contigs, to speed up the process. Results from RepeatMasker are then summarised using in-house python script.

Code availability

All scripts used to generate the assembly are available on GitHub
https://github.com/evotools/CattleGraphGenomePaper/tree/master/Assembly/ANK1

Supplementary Note 3 – Collection of figures describing the quality metrics of variants called using FreeBayes, GATK4, VG on a linear graph (VG1), VG on a graph with 11M variants from Dutta et al 2020 (VG1p), VG on a CACTUS-derived graph incorporating 5 different assemblies, VG on the VG5 graph expanded with the 11M variants included in VG1p (VG5p).

Number of variants

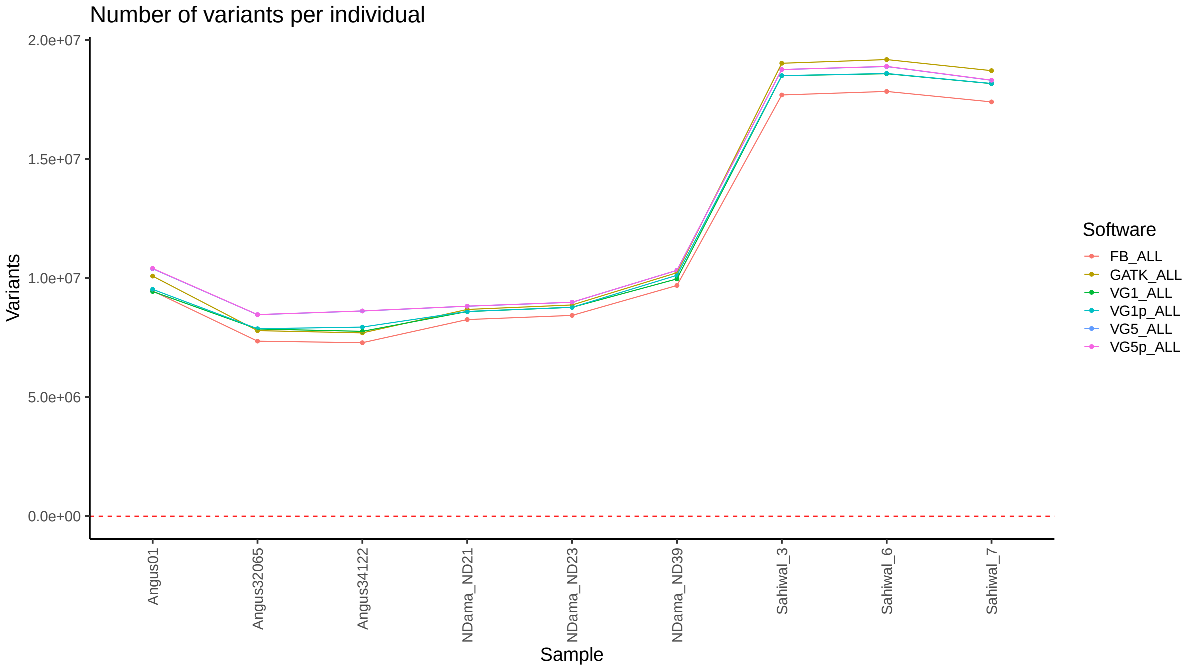

Variant number by individual and algorithm

ALGORITHM	SIZE	Angus01	Angus<br>Angus32065	Angus34122	NDama_ND21	N'Dama<br>NDama_ND23	NDama_ND39	Sahiwal_3	Sahiwal<br>Sahiwal_6	Sahiwal_7
	0	9,433,407	8,680,825	8,647,275	10,293,619	10,427,815	11,365,489	22,267,905	21,961,629	21,134,702
	30	1,247,675	1,291,245	1,286,982	1,459,222	1,465,023	1,528,898	2,601,979	2,563,991	2,465,586
	100	699	978	912	5,096	5,147	5,258	11,094	10,589	10,292
	500	0	0	0	0	0	0	0	0	0
	1000	0	0	0	0	0	0	0	0	0
	5000	0	0	0	0	0	0	0	0	0
FB	>=5001	0	0	0	0	0	0	0	0	0
	0	9,714,748	9,122,613	9,062,461	10,781,189	10,925,493	11,938,304	23,933,608	23,600,031	22,705,447
	30	1,410,833	1,390,929	1,365,907	1,648,530	1,658,077	1,741,077	2,916,412	2,880,400	2,791,150
	100	11,414	13,255	12,474	18,693	18,947	20,196	38,075	37,585	36,361
	500	598	829	731	2,040	2,007	2,082	3,840	3,609	3,593
	1000	0	0	0	0	0	0	0	0	0
	5000	0	0	0	0	0	0	0	0	0
GATK	>=5001	0	0	0	0	0	0	0	0	0
	0	9,310,979	9,146,153	8,982,935	10,637,904	10,779,804	11,644,564	23,176,834	22,793,550	21,995,140
	30	1,202,143	1,200,398	1,180,741	1,403,965	1,413,178	1,499,336	2,515,032	2,495,894	2,409,959
	100	503	753	623	3,521	3,587	3,611	7,785	7,263	7,530
	500	0	0	0	0	0	0	0	0	0
	1000	0	0	0	0	0	0	0	0	0
	5000	0	0	0	0	0	0	0	0	0
VG1	>=5001	0	0	0	0	0	0	0	0	0
	0	9,426,559	9,161,053	9,195,152	10,642,495	10,782,819	11,803,691	23,192,625	22,817,076	22,009,622
	30	1,202,013	1,203,250	1,207,983	1,406,005	1,415,432	1,504,931	2,519,744	2,500,724	2,413,828
	100	552	814	685	3,580	3,620	3,851	7,940	7,421	7,664
	500	8	8	3	6	6	5	12	13	12
	1000	0	0	0	0	0	0	0	0	0
	5000	0	0	0	0	0	0	0	0	0
VG1P	>=5001	0	0	0	0	0	0	0	0	0
	0	10,384,667	9,830,558	9,956,794	10,963,951	11,081,224	12,099,440	23,604,880	23,263,468	22,289,674
	30	1,362,140	1,350,017	1,363,988	1,564,503	1,575,635	1,683,092	2,786,138	2,767,011	2,650,831
	100	14,155	14,868	14,089	17,711	17,992	19,057	32,716	32,308	31,196
	500	8,947	8,943	8,665	10,028	10,119	10,739	16,620	16,619	16,134
	1000	2,632	2,510	2,475	2,607	2,610	2,858	4,279	4,208	4,089
	5000	3,357	3,266	3,227	3,317	3,268	3,550	4,981	4,943	4,871
VG5	>=5001	343	340	341	387	359	382	447	436	443
	0	10,404,632	9,834,954	9,961,919	10,968,870	11,084,581	12,101,691	23,613,489	23,274,904	22,299,958
	30	1,361,051	1,349,330	1,362,827	1,563,403	1,574,625	1,681,623	2,782,881	2,764,108	2,647,688
	100	14,128	14,865	14,117	17,731	17,984	19,023	32,740	32,317	31,201
	500	8,962	8,941	8,655	10,030	10,105	10,724	16,558	16,580	16,095
	1000	2,619	2,506	2,475	2,617	2,605	2,853	4,259	4,187	4,085
	5000	3,368	3,230	3,218	3,299	3,268	3,542	4,931	4,939	4,834
VG5P	>=5001	345	347	341	386	356	377	449	437	444

Scenario A: 11M variants as known

Variant number by size

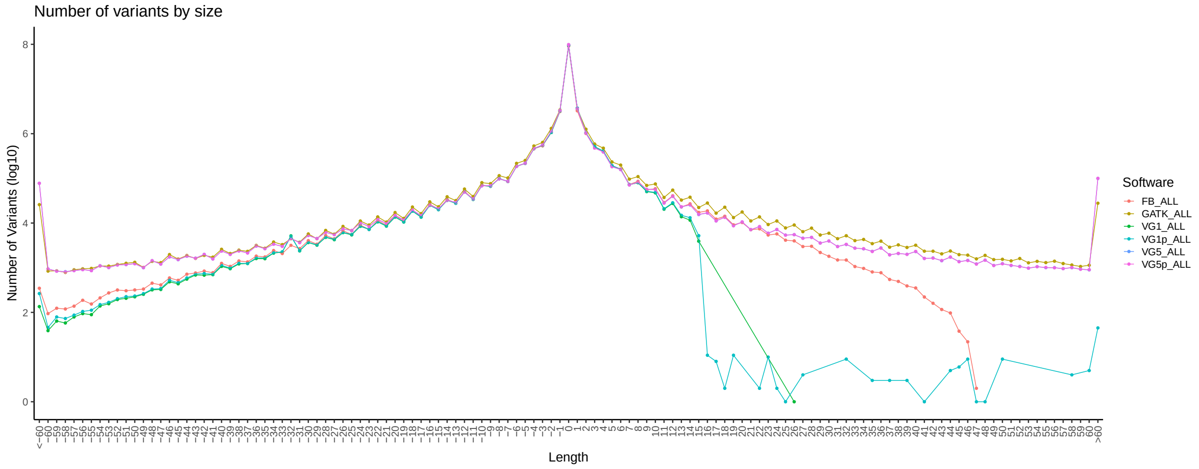

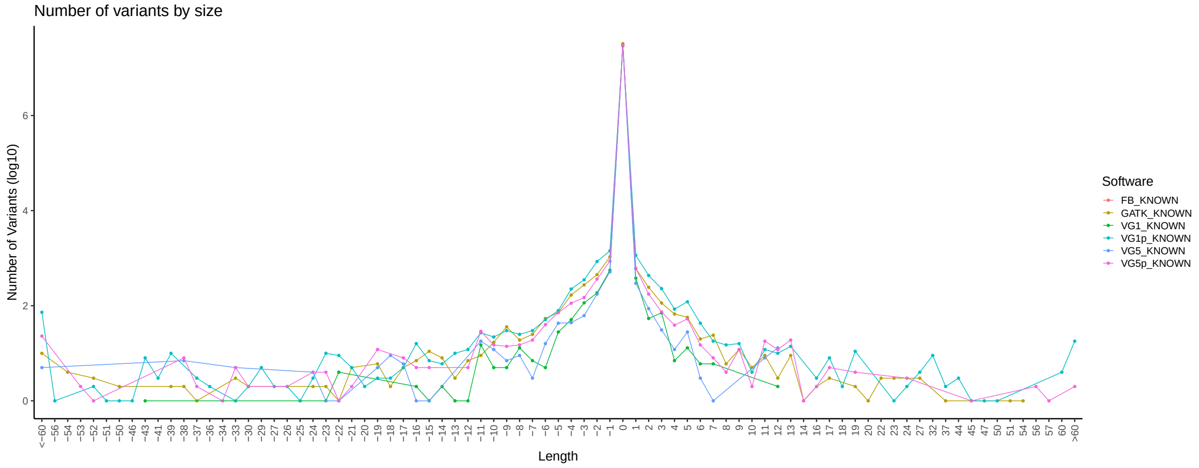

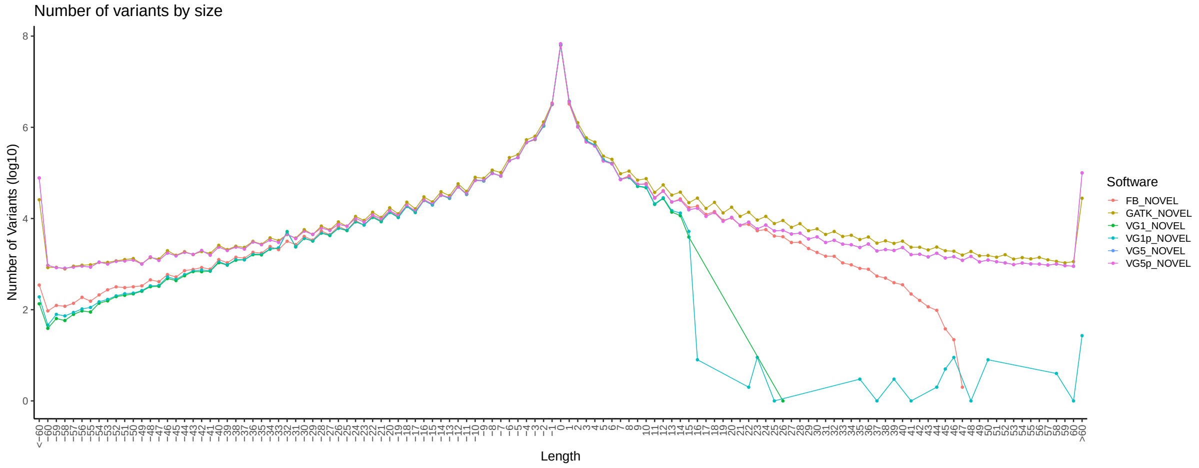

Variant QUAL by Size

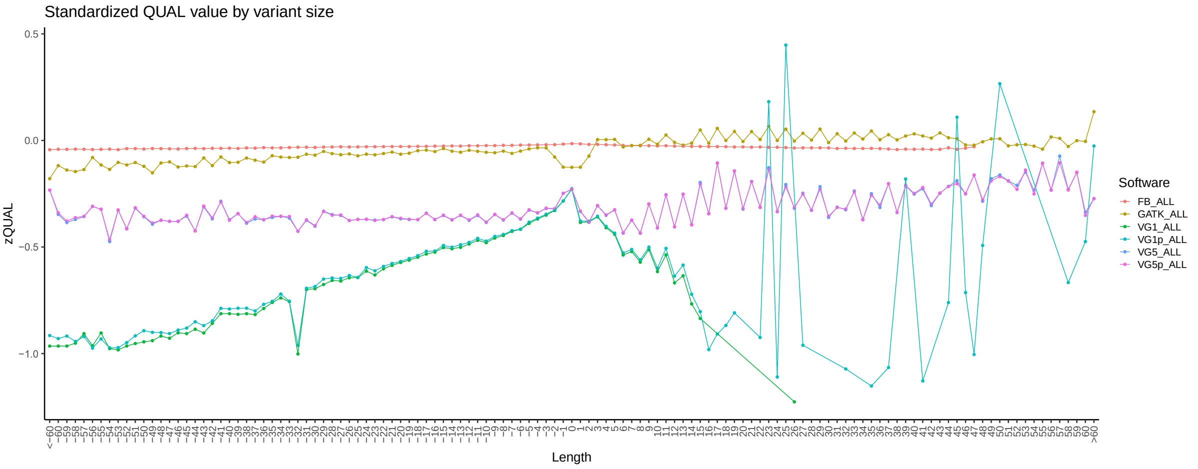

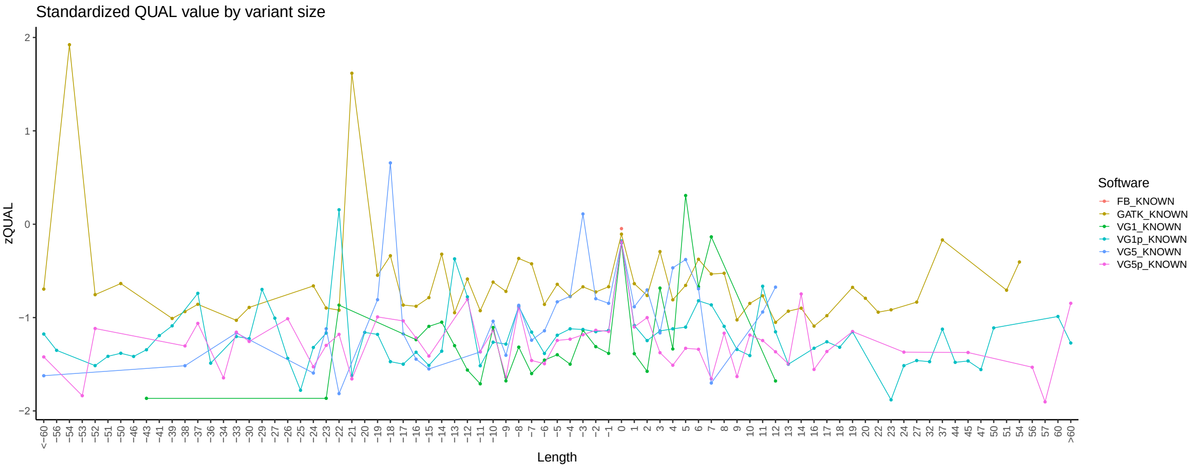

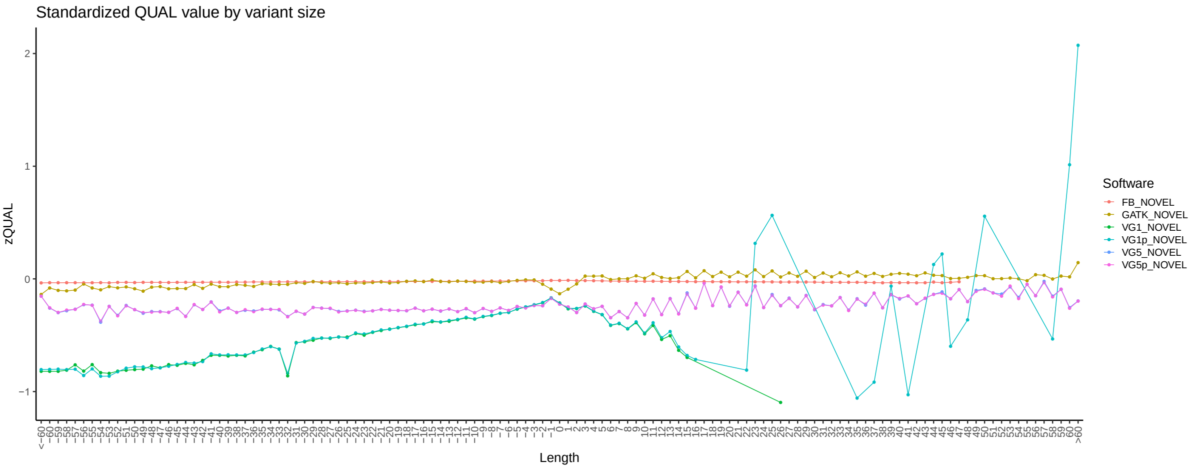

Variant Depth by Size

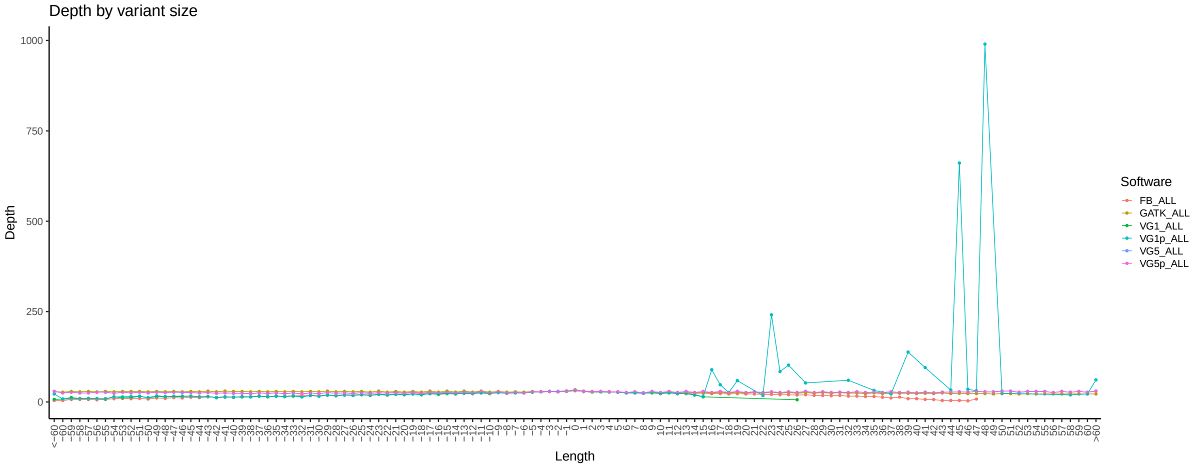

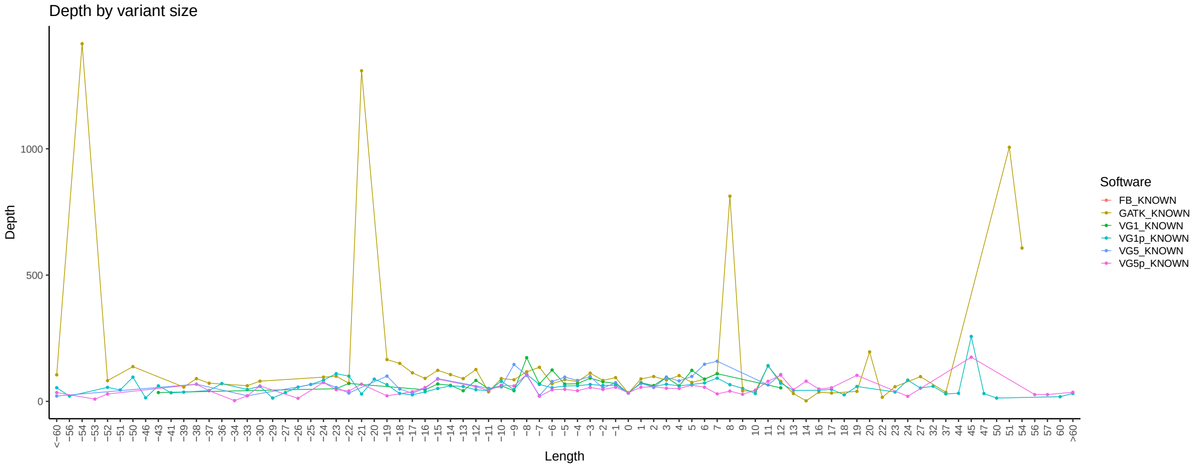

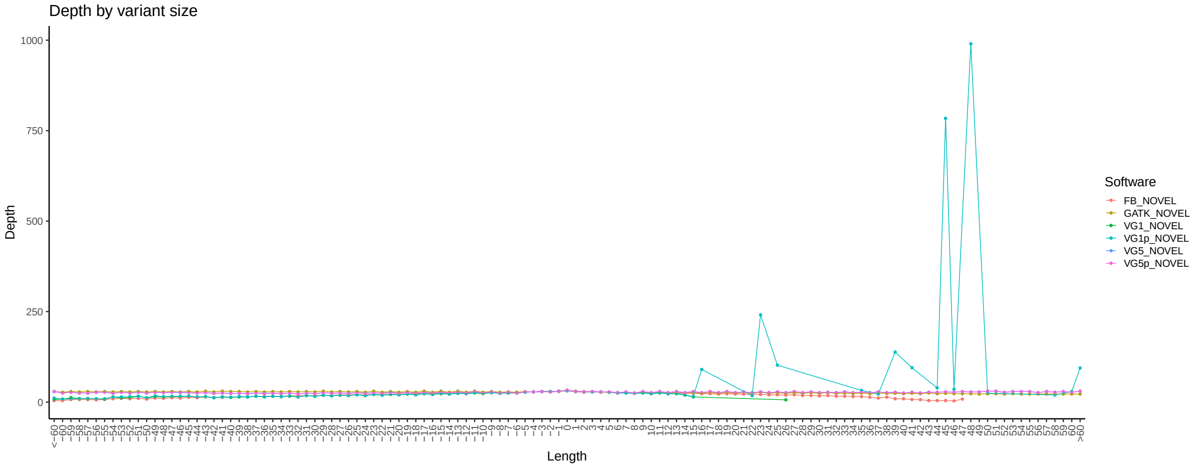

Variant Allelic Balance (AB)

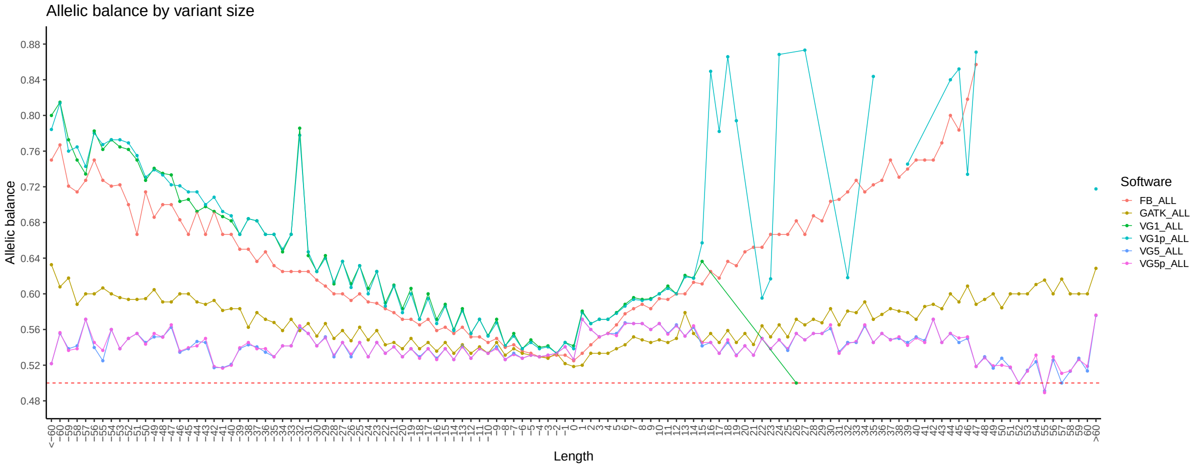

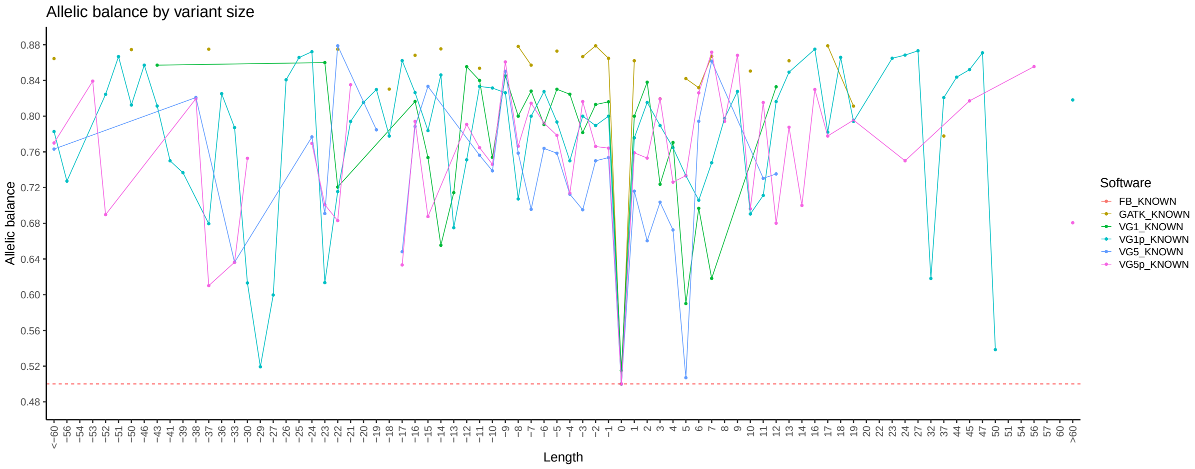

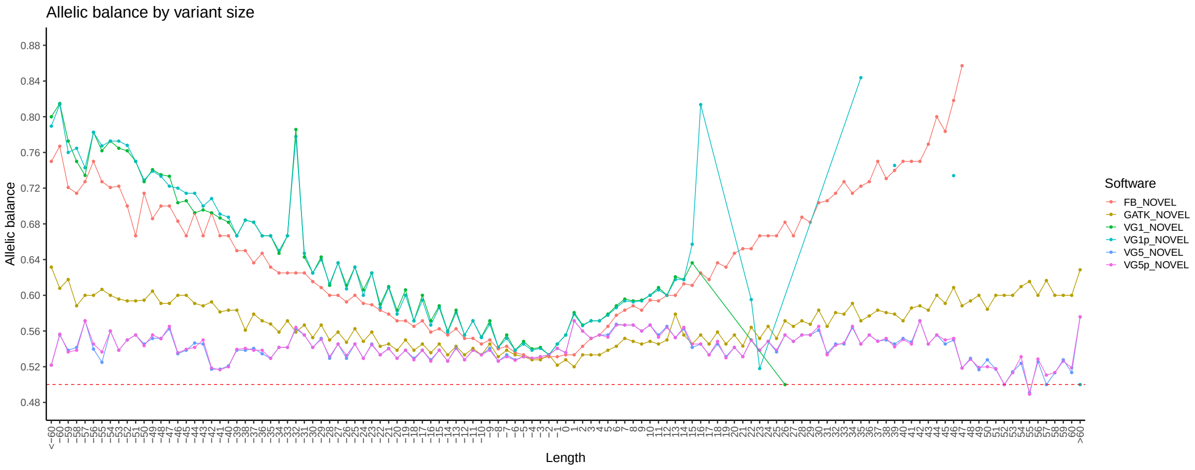

Variant Transition/Transversion ratio (TiTv)

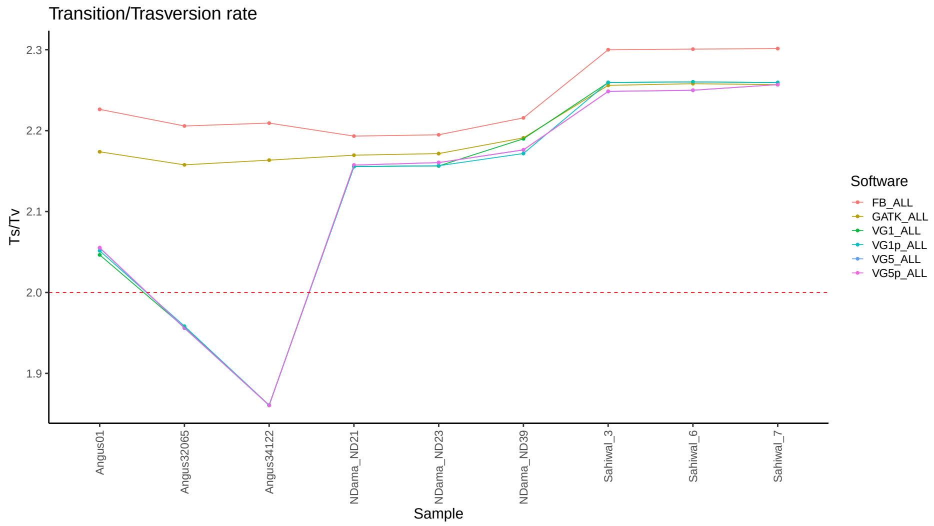

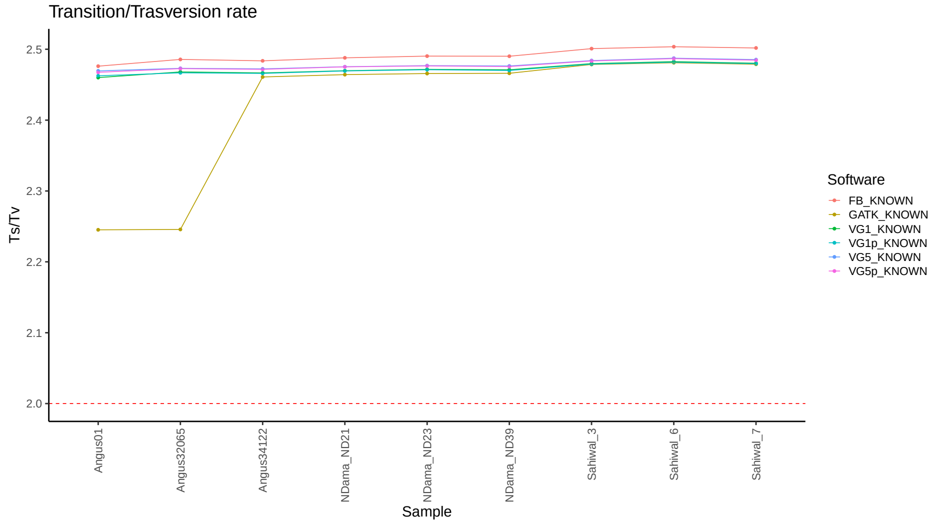

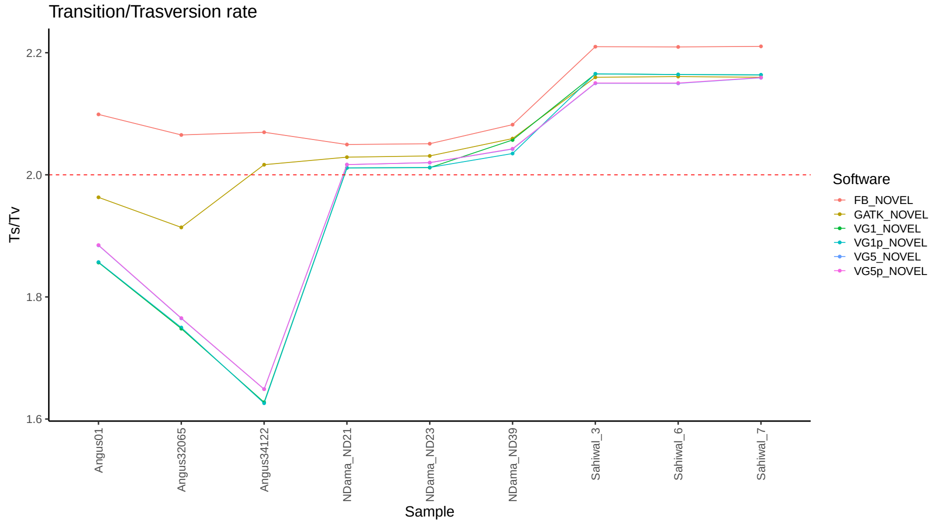

Scenario B: Graph-specific variants as known

Variant Number by size

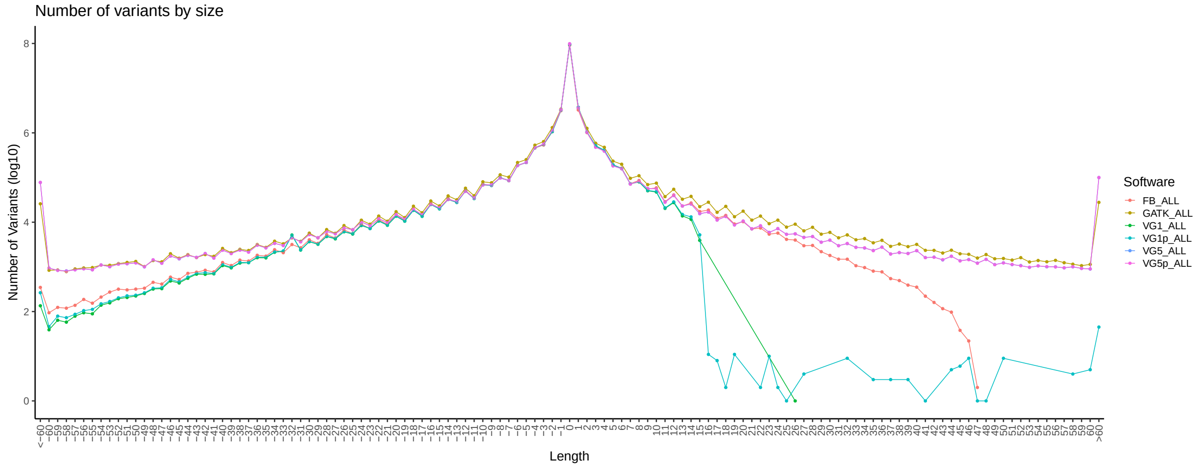

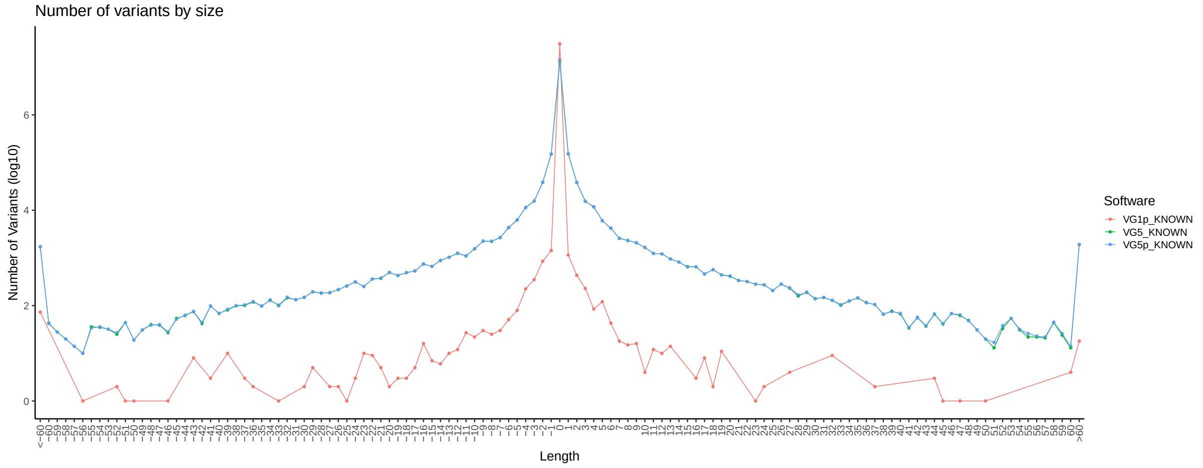

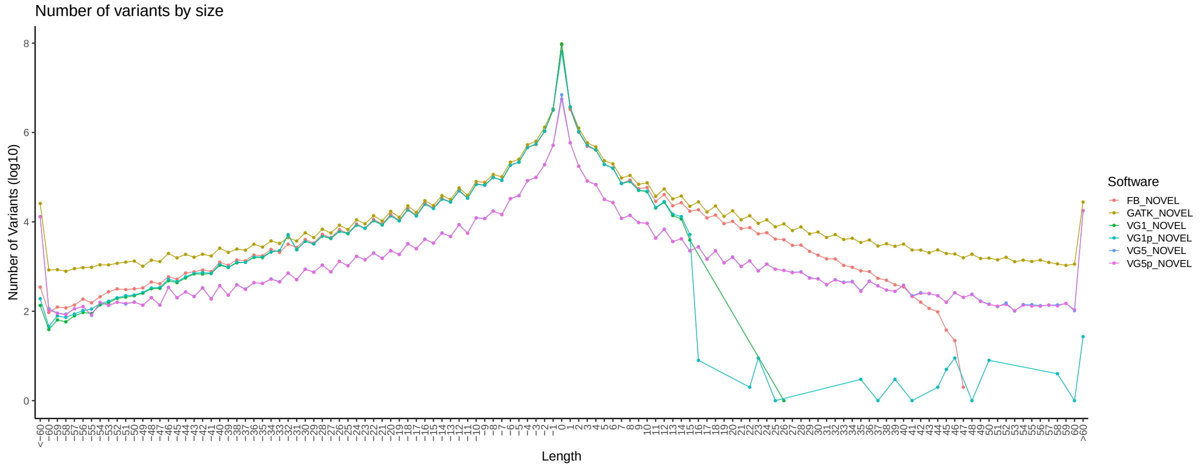

Variant QUAL by Size

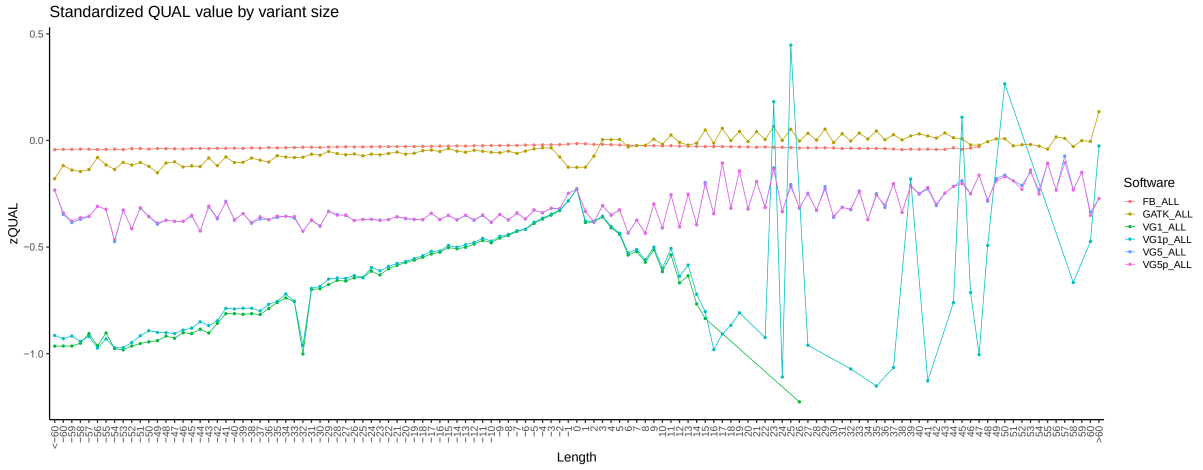

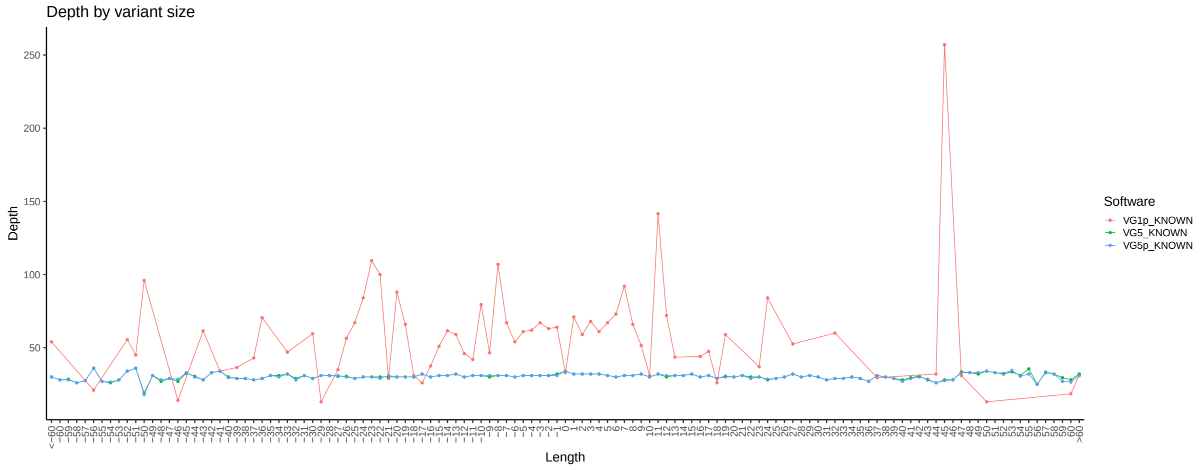

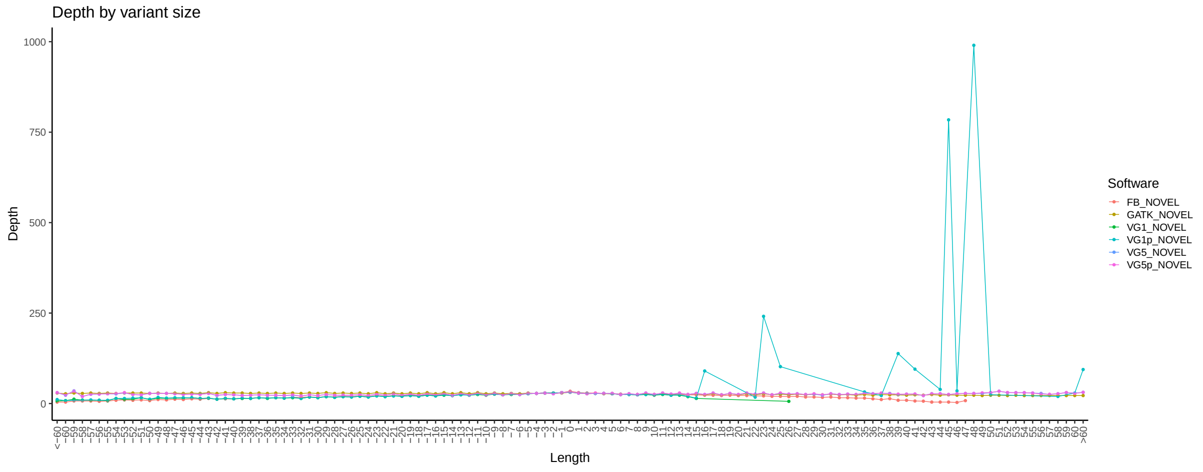

Variant Depth by Size

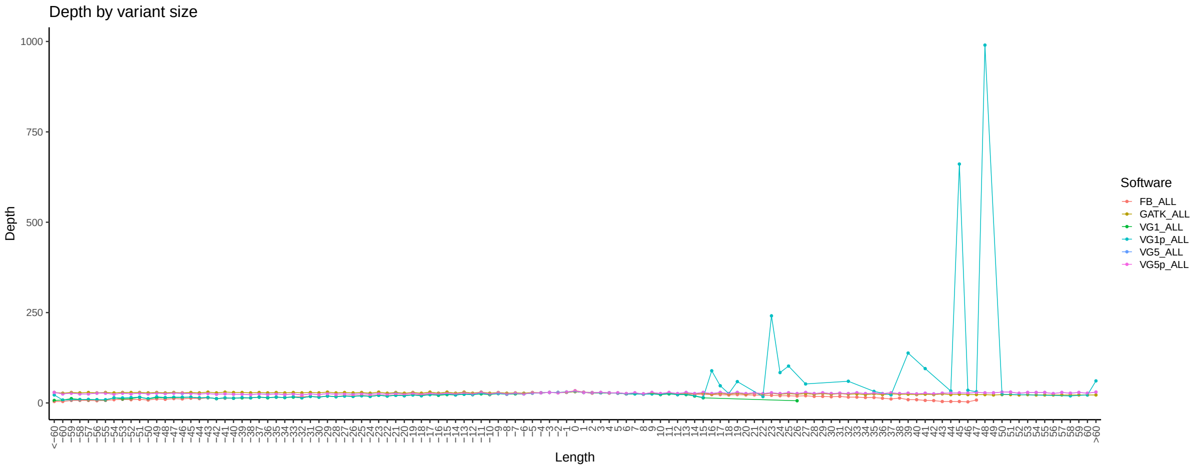

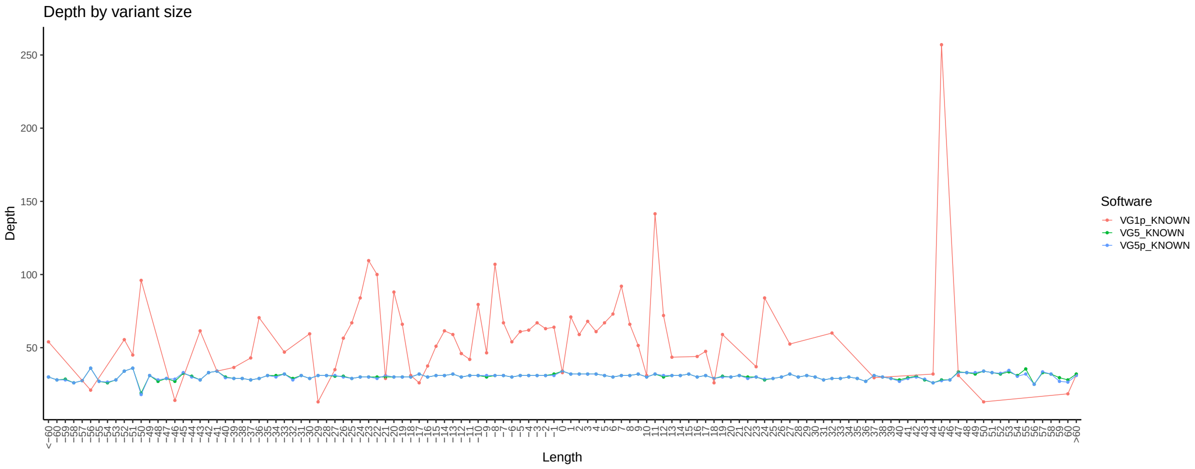

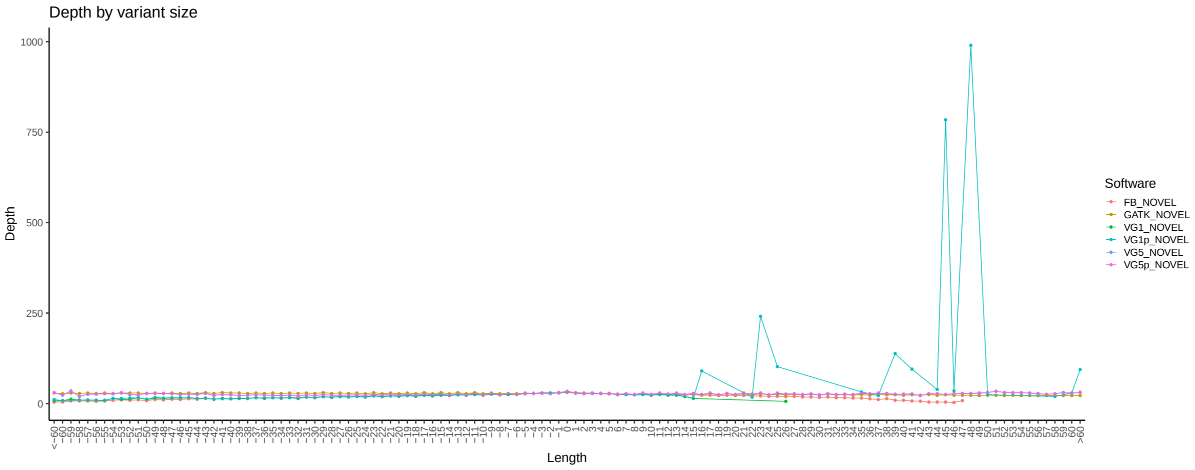

Variant Allelic Balance (AB) by Size

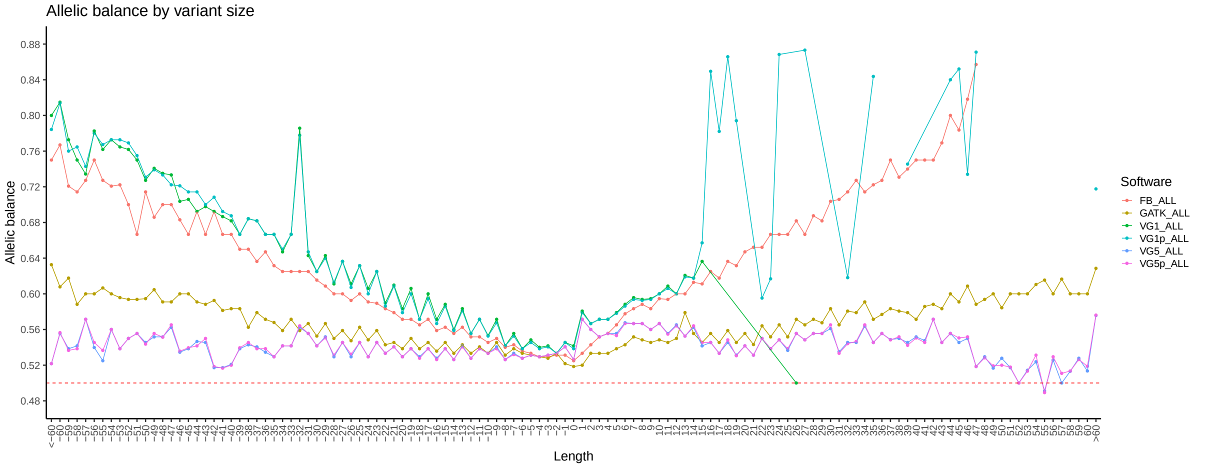

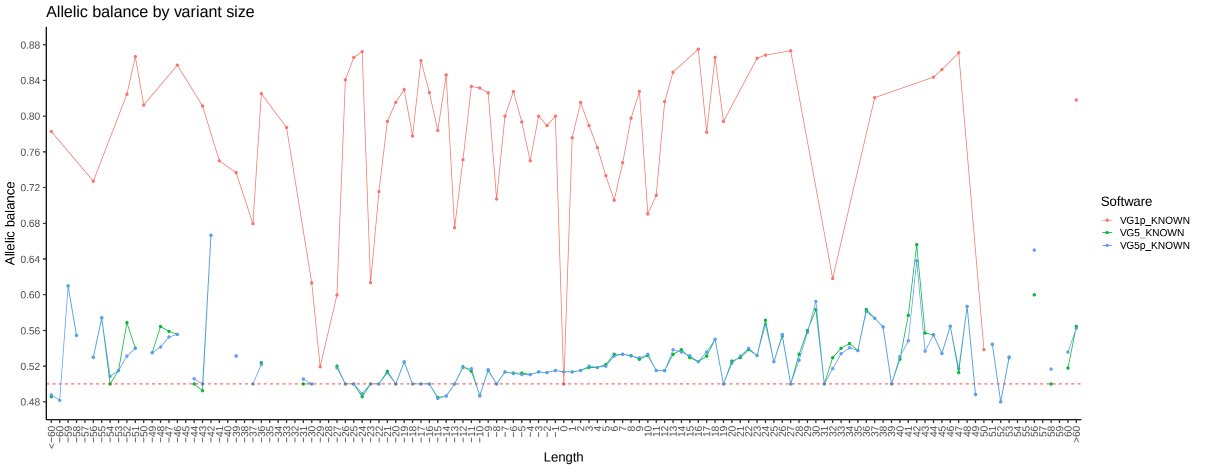

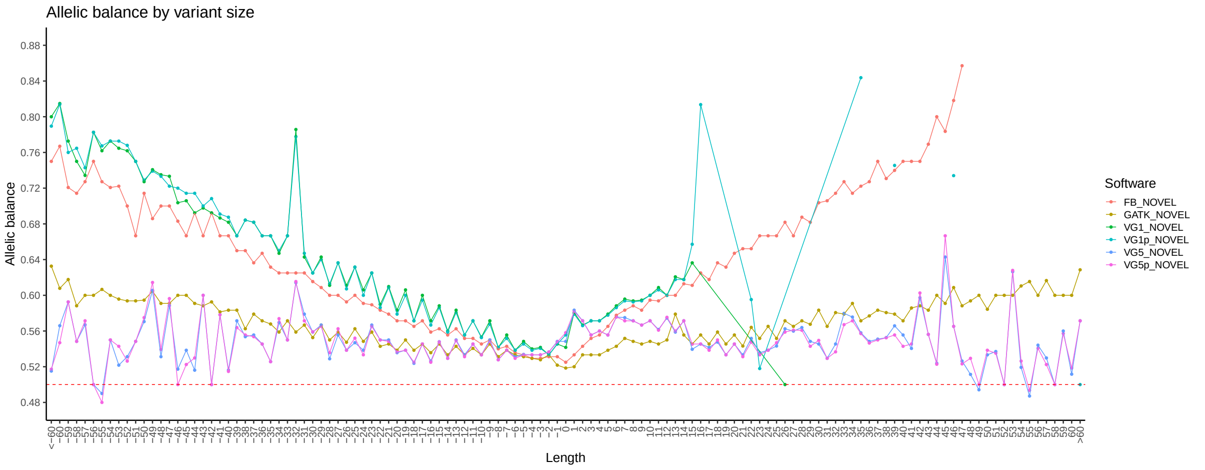

Variant Transition/Transversion ratio (TiTv)

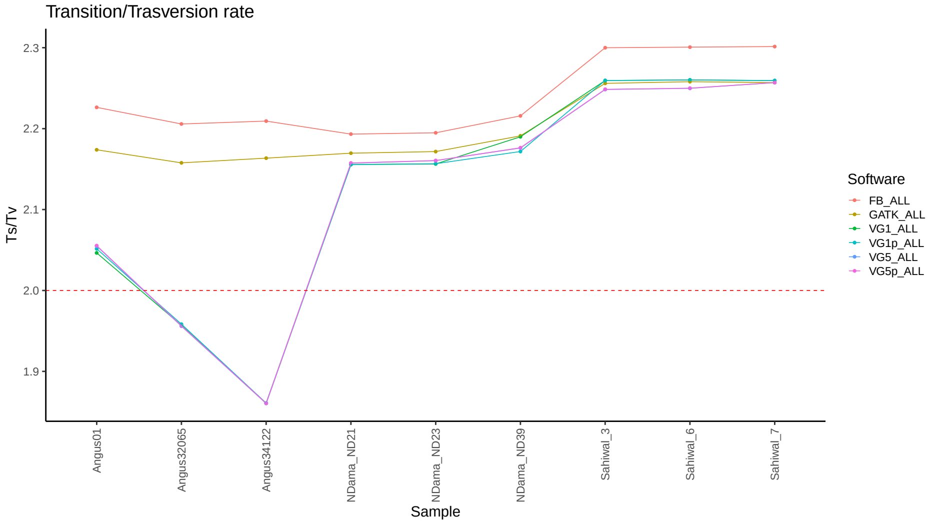

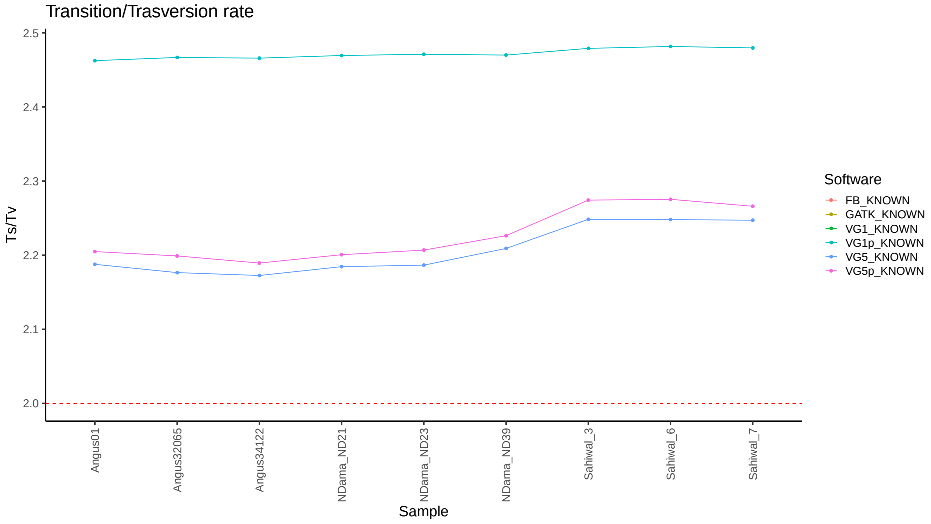

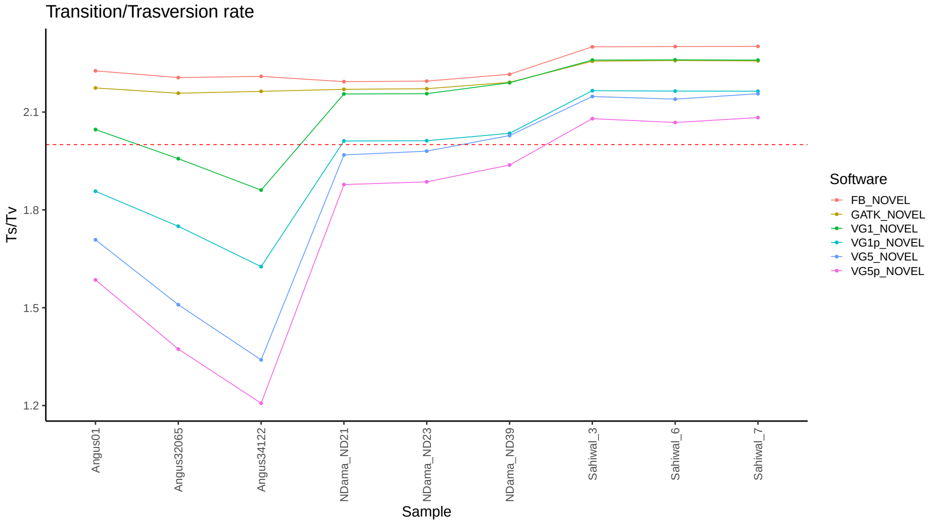

Supplementary Tables

Supplementary Table 1: Nodes (i.e. fragments of sequence), edges (connections between nodes) and lengths for the four graph genomes generated using VG.

	Nodes				Edges				Length			
Chromosome	VG1	VG1p	VG5	VG5P	VG1	VG1p	VG5	VG5P	VG1	VG1p	VG5	VG5P
1	4,954,191	6,584,429	9,251,514	10,096,305	4,954,190	7,261,296	10,843,437	11,987,980	158,534,110	159,193,840	163,993,460	164,296,320
2	4,257,222	5,703,869	7,789,362	8,555,899	4,257,221	6,304,205	9,101,550	10,139,634	136,231,102	136,815,897	140,416,456	140,690,580
3	3,781,412	5,151,300	6,867,567	7,656,465	3,781,411	5,720,530	8,011,124	9,078,791	121,005,158	121,559,594	125,147,658	125,429,553
4	3,750,019	5,111,687	6,953,975	7,714,659	3,750,018	5,677,859	8,150,173	9,181,067	120,000,601	120,552,785	123,711,581	123,985,996
5	3,752,792	4,999,817	7,129,985	7,837,366	3,752,791	5,518,011	8,330,515	9,288,043	120,089,316	120,593,886	129,150,326	129,403,184
6	3,681,449	4,875,026	6,937,331	7,561,643	3,681,448	5,370,944	8,140,751	8,986,587	117,806,340	118,288,741	121,820,029	122,043,625
7	3,458,836	4,611,447	6,483,931	7,071,049	3,458,835	5,090,341	7,601,548	8,396,963	110,682,743	111,149,259	115,094,870	115,305,592
8	3,541,243	4,813,024	6,474,859	7,195,986	3,541,242	5,341,227	7,558,963	8,535,175	113,319,770	113,833,971	117,751,945	118,009,353
9	3,295,453	4,363,489	6,519,015	7,094,968	3,295,452	4,807,291	7,610,124	8,390,406	105,454,467	105,886,239	117,321,810	117,528,044
10	3,228,399	4,492,980	6,274,280	6,975,100	3,228,398	5,018,764	7,332,030	8,281,374	103,308,737	103,820,804	111,960,534	112,212,201
11	3,343,203	4,511,925	6,006,286	6,670,888	3,343,202	4,997,621	6,994,503	7,894,020	106,982,474	107,455,061	110,063,050	110,300,342
12	2,725,506	3,579,705	6,325,281	6,799,943	2,725,505	3,934,631	7,582,330	8,226,150	87,216,183	87,562,591	101,279,991	101,451,234
13	2,608,511	3,694,577	4,713,442	5,343,381	2,608,510	4,146,392	5,489,335	6,342,511	83,472,345	83,911,288	86,400,371	86,625,722
14	2,575,094	3,490,845	4,746,257	5,255,609	2,575,093	3,871,460	5,515,104	6,204,440	82,403,003	82,773,593	87,891,652	88,073,781
15	2,656,494	3,645,440	5,439,455	5,973,670	2,656,493	4,056,762	6,442,808	7,166,965	85,007,780	85,408,361	91,227,610	91,420,305
16	2,531,687	3,443,457	4,709,468	5,221,595	2,531,686	3,822,266	5,502,216	6,195,675	81,013,979	81,382,579	84,645,483	84,828,625
17	2,286,477	3,055,545	4,274,504	4,705,739	2,286,476	3,375,203	5,007,621	5,591,619	73,167,244	73,478,087	76,304,198	76,458,463
18	2,056,895	2,770,605	3,890,050	4,322,813	2,056,894	3,067,595	4,553,450	5,139,526	65,820,629	66,109,525	69,204,425	69,359,413
19	1,982,805	2,659,019	3,750,778	4,115,143	1,982,804	2,939,942	4,403,051	4,896,405	63,449,741	63,723,150	66,113,060	66,243,432
20	2,249,207	3,047,288	3,937,801	4,428,948	2,249,206	3,379,179	4,556,671	5,221,477	71,974,595	72,297,013	74,993,590	75,168,771
21	2,183,218	2,951,323	4,268,389	4,687,235	2,183,217	3,271,024	5,026,387	5,594,312	69,862,954	70,173,574	74,009,075	74,159,834
22	1,899,158	2,699,988	3,436,590	3,914,393	1,899,157	3,033,041	3,995,583	4,642,422	60,773,035	61,096,239	63,369,249	63,539,916
23	1,640,582	2,365,162	3,574,698	3,986,677	1,640,581	2,667,044	4,272,809	4,831,633	52,498,615	52,791,725	57,079,504	57,228,272
24	1,947,415	2,696,224	3,696,008	4,090,885	1,947,414	3,007,528	4,348,584	4,883,751	62,317,253	62,619,389	64,016,876	64,158,252
25	1,323,452	1,786,548	2,383,272	2,646,103	1,323,451	1,979,151	2,776,810	3,133,038	42,350,435	42,537,360	43,569,786	43,663,965
26	1,624,760	2,251,109	2,958,553	3,331,871	1,624,759	2,511,443	3,456,680	3,961,983	51,992,305	52,245,403	53,708,772	53,842,318
27	1,425,379	1,991,336	2,883,714	3,187,086	1,425,378	2,227,426	3,419,790	3,831,449	45,612,108	45,840,707	48,137,849	48,247,190
28	1,435,630	2,038,693	2,856,754	3,209,997	1,435,629	2,289,739	3,324,155	3,802,741	45,940,150	46,183,254	52,653,916	52,780,354
29	1,596,832	2,192,891	3,209,231	3,526,945	1,596,831	2,441,034	3,795,304	4,225,973	51,098,607	51,339,594	54,311,770	54,426,115
Total	77,793,321	105,578,748	147,742,350	163,178,361	77,793,292	117,128,949	173,143,406	194,052,110	2,489,385,779	2,500,623,509	2,625,348,896	2,630,880,752

Supplementary Table 2: Number of structural variants detected using the VG5p graph on all samples and those specific to the different breeds, with the number of overlaps with variants from optical mapping in comparison of 10,000 random regions of equal size and respective two-tailed P values calculated from the Z-scores.

	GLOBAL SV (VG5p)	ANGUS SV (VG5p)	NDAMA SV (VG5p)	SAHIWAL SV (VG5p)
Overlap with optical mapping SV	6598	10	42	111
Total SVs considered	12306	19	49	299
Random SV tests	10000	10000	10000	10000
Mean	1571.1736	2.5814	6.2295	38.5017
StD	36.92566335	1.32088929	2.34400921	5.790177
Z-score	136.133679	5.61636775	15.2603922	12.5209126
P-value	0	1.9501E-08	1.404E-52	5.7372E-36

Supplementary Table 3: Number of structural variants from the VG5p graph longer than 500 bp and those overlapping an optical mapping SV.

Breed	SV>500bp	SV>500bp in OM	Ratio	Average size
Angus	7318	2797	38.22	2050.66
NDama	7280	2932	40.27	2055.42
Sahiwal	10046	3368	33.53	1880.9

Supplementary table 4: Number of structural variants discovered using DellyV2 at the different filtering stages.

Breed	Sample	N SV	Filtered SVs genotyped (individual)	500bp SV by breed	500bp SV dataset	500bp SV dataset overlapping optical maps	500bp SV dataset overlapping optical maps (%ge)
Angus	Angus01	7,533	2,167	3,175	11,562	5,371	46.45%
	Angus32065	7,244	2,008				
	Angus34122	6,878	1,940				
N'Dama	ND21	15,061	2,945	5,206			
	ND23	15,474	3,010				
	ND39	17,399	3,418				
Sahiwal	Sahiwal_3	30,941	5,395	8,421			
	Sahiwal_6	31,162	5,396				
	Sahiwal_7	30,466	5,356				

Supplementary Table 5: Number of ATAC-seq reads mapped to the different linear, breed-specific genomes and to the expanded linear Hereford genome (ARS-UCD1.2+), with the relative improvement in the latter in comparison with the standard Hereford genome.

Breed	Sample	TOTAL	ANGUS	ANKOLE	BRAHMAN	HEREFORD	NDAMA	ARS-UCD1.2+	Improvement
Holstein	HF3457 (B-cell)	443,051,164	567,930,948	465,623,093	457,627,237	451,083,749	484,590,179	469,621,291	1.041095566
N'Dama	ND230 (B-cell)	625,474,292	773,090,861	654,307,023	642,870,385	632,982,181	678,181,434	658,824,369	1.040826091
Nelore	Nelore2 (B-cell)	1,773,894,312	2,425,549,286	1,934,181,179	1,861,955,997	1,848,420,148	2,022,532,403	1,922,158,212	1.039892480
Holstein	HF3457 (nucleosome-free)	1,169,362,448	1,186,338,288	1,178,742,664	1,170,695,333	1,156,154,503	1,189,296,521	1,180,022,346	NA

Supplementary Table 6: Peaks called using the different linear, breed-specific assemblies and the expanded linear Hereford genome (ARS-UCD1.2+), with the number of peaks after excluding the signals in common with the nuclease-free peaks and the number overlapping a predicted gene from Augustus.

Reference	Breed	Sample	Raw	No blank	No blank significant	Low repetitive content	Low repetitive content significant	Cross-referenced	Ratio	Ratio significant
ANGUS	Holstein	HF3457_Bcell_ATAC	37811	33096	26	24468	22	0	0.845	0.813
ANGUS	N'Dama	ND230_Bcell_ATAC	37768	34397	39	22827	34	0	0.734	0.796
ANGUS	Nelore	Nelore2_Bcell_ATAC	55209	50266	722	33454	701	0	0.886	0.899
ANKOLE	Holstein	HF3457_Bcell_ATAC	47367	38177	74	24242	14	0	0.975	2.313
ANKOLE	N'Dama	ND230_Bcell_ATAC	46388	40166	124	22603	14	0	0.857	2.531
ANKOLE	Nelore	Nelore2_Bcell_ATAC	65189	55723	782	34384	680	0	0.982	0.974
ARS-UCD1.2+	Holstein	HF3457_Bcell_ATAC	45041	40621	19	29581	15	38396	1.037	0.594
ARS-UCD1.2+	N'Dama	ND230_Bcell_ATAC	50728	47608	30	25637	24	44390	1.016	0.612
ARS-UCD1.2+	Nelore	Nelore2_Bcell_ATAC	60841	57451	832	35226	801	55408	1.013	1.036
BRAHMAN	Holstein	HF3457_Bcell_ATAC	36426	33216	20	26484	14	0	0.848	0.625
BRAHMAN	N'Dama	ND230_Bcell_ATAC	40204	37672	28	25871	18	0	0.804	0.571
BRAHMAN	Nelore	Nelore2_Bcell_ATAC	51011	48965	742	34228	709	0	0.863	0.924
HEREFORD	Holstein	HF3457_Bcell_ATAC	43464	39160	32	28524	28	0	1.000	1.000
HEREFORD	N'Dama	ND230_Bcell_ATAC	49826	46857	49	25271	43	0	1.000	1.000
HEREFORD	Nelore	Nelore2_Bcell_ATAC	59961	56717	803	34970	772	0	1.000	1.000
NDAMA	Holstein	HF3457_Bcell_ATAC	45679	41078	47	29602	32	0	1.049	1.469
NDAMA	N'Dama	ND230_Bcell_ATAC	51818	48779	77	25907	57	0	1.041	1.571
NDAMA	Nelore	Nelore2_Bcell_ATAC	57639	54814	869	35242	834	0	0.966	1.082
HEREFORD_unique	Holstein	HF3457_Bcell_ATAC	146177	145020	36	117089	36	0	1.000	1.000
HEREFORD_unique	N'Dama	ND230_Bcell_ATAC	205374	204361	322	154885	321	0	1.000	1.000
HEREFORD_unique	Nelore	Nelore2_Bcell_ATAC	73084	72463	2849	55329	2703	0	1.000	1.000
ARS-UCD1.2+_unique	Holstein	HF3457_Bcell_ATAC	149386	148426	39	119525	39	0	1.023	1.083
ARS-UCD1.2+_unique	N'Dama	ND230_Bcell_ATAC	210319	209480	320	158348	319	0	1.025	0.994
ARS-UCD1.2+_unique	Nelore	Nelore2_Bcell_ATAC	73751	73272	2873	55907	2725	0	1.011	1.008

Supplementary Table 7: List of samples used in the study, with their associated accessions.

Type	Sample ID	Sample ENA	Library	Base yield	Coverage (x)	Platform
WGS	Angus01	SAMN03387020	SRR2016763	30,243,902,200	34.38	Illumina HiSeq 2000
			SRR2016765	23,263,065,800		Illumina HiSeq 2000
			SRR2016766	15,033,757,200		Illumina HiSeq 2000
			SRR2016767	24,278,048,000		Illumina HiSeq 2000
WGS	Angus32065	SAMN05788510	SRR4280084	50,893,456,010	33.42	Illumina HiSeq 2000
			SRR4280085	39,338,730,967		Illumina HiSeq 2000
WGS	Angus34122	SAMN05788530	SRR4280168	47,012,045,232	31.85	Illumina HiSeq 2500
			SRR4280169	38,995,161,038		Illumina HiSeq 2500
WGS	NDama_ND21	ERS4826976	ERS4826976	139,358,632,563	51.61	HiSeq X Ten
WGS	NDama_ND23	ERS4826977	ERR4352199	142,322,660,295	52.71	HiSeq X Ten
WGS	NDama_ND39	ERS4826978	ERR4352264	142,976,110,300	52.95	HiSeq X Ten
WGS	Sahiwal_3	ERS4818261	ERR4350687	125,539,224,218	46.50	HiSeq X Ten
WGS	Sahiwal_6	ERS4818262	ERR4336349	120,710,854,101	44.71	HiSeq X Ten
WGS	Sahiwal_7	ERS4818263	ERR4336437	128,921,757,833	47.75	HiSeq X Ten
ATAC	HF3457_Bcell_ATAC	ERS8971208	ERR7448822			Illumina HiSeq 4000
ATAC			ERR7448819			Illumina HiSeq 4000
ATAC			ERR7448818			Illumina HiSeq 4000
ATAC			ERR7448820			Illumina HiSeq 4000
ATAC	ND230_Bcell_ATAC	ERS8971209	ERR7451279			Illumina NovaSeq 6000
ATAC	Nelore2_Bcell_ATAC	ERS8971210	ERR7451506			Illumina NovaSeq 6000
ATAC			ERR7451524			Illumina NovaSeq 6000
ATAC	HF3457_nucfree_ATAC	ERS8971208	ERR7448832			Illumina HiSeq 4000
ATAC			ERR7448837			Illumina HiSeq 4000
ATAC			ERR7448839			Illumina HiSeq 4000
ATAC			ERR7448835			Illumina HiSeq 4000
WGS	Angus_SAMN05788481		E:SRR4279946			Illumina HiSeq 2500
WGS	Angus_SAMN05788481		E:SRR4279947			Illumina HiSeq 2500
WGS	Angus_SAMN05788481		E:SRR4279948			Illumina HiSeq 2500
WGS	Angus_SAMN05788481		E:SRR4279949			Illumina HiSeq 2500
WGS	Angus_SAMN05788480		E:SRR4279940			Illumina HiSeq 2500
WGS	Angus_SAMN05788480		E:SRR4279941			Illumina HiSeq 2500
WGS	Angus_SAMN05788480		E:SRR4279942			Illumina HiSeq 2500
WGS	Angus_SAMN05788480		E:SRR4279943			Illumina HiSeq 2500
WGS	Angus_SAMN05788480		E:SRR4279944			Illumina HiSeq 2500
WGS	Angus_SAMN05788480		E:SRR4279945			Illumina HiSeq 2500
WGS	Angus_SAMN05788482		E:SRR4279950			Illumina HiSeq 2500
WGS	Angus_SAMN05788482		E:SRR4279951			Illumina HiSeq 2500
WGS	Angus_SAMN05788482		E:SRR4279952			Illumina HiSeq 2500
WGS	Angus_SAMN05788482		E:SRR4279953			Illumina HiSeq 2500
OM	Ndama_1_1628_A	ERS8452869	ERZ4193982			Bionano Saphyr
OM	Ndama_NN031_B	ERS8452868	ERZ4193983			Bionano Saphyr

Supplementary references

1. Buenrostro, J. D. *et al.* Single-cell chromatin accessibility reveals principles of regulatory variation. *Nature* (2015) doi:10.1038/nature14590.
2. Li, H. Minimap2: Pairwise alignment for nucleotide sequences. *Bioinformatics* **34**, 3094–3100 (2018).
3. Li, H. *et al.* The Sequence Alignment/Map format and SAMtools. *Bioinformatics* **25**, 2078–2079 (2009).
4. Koren, S. *et al.* Canu: Scalable and accurate long-read assembly via adaptive κ-mer weighting and repeat separation. *Genome Res.* **27**, 722–736 (2017).
5. Chin, C. S. *et al.* Phased diploid genome assembly with single-molecule real-time sequencing. *Nat. Methods* **13**, 1050–1054 (2016).
6. Walker, B. J. *et al.* Pilon: An integrated tool for comprehensive microbial variant detection and genome assembly improvement. *PLoS ONE* **9**, e112963–e112963 (2014).
7. Waterhouse, R. M. *et al.* BUSCO Applications from Quality Assessments to Gene Prediction and Phylogenomics. *Mol. Biol. Evol.* **35**, 543–548 (2018).
8. Minkin, I. & Medvedev, P. Scalable multiple whole-genome alignment and locally collinear block construction with SibeliaZ. *bioRxiv* 548123–548123 (2019) doi:10.1101/548123.
9. Kolmogorov, M. *et al.* Chromosome assembly of large and complex genomes using multiple references. *Genome Res.* **28**, 1720–1732 (2018).
10. Mikheenko, A., Prjibelski, A., Saveliev, V., Antipov, D. & Gurevich, A. Versatile genome assembly evaluation with QUAST-LG. in vol. 34 i142–i150 (Oxford University Press, 2018).
11. Xu, G.-C. *et al.* LR_Gapcloser: a tiling path-based gap closer that uses long reads to complete genome assembly. *GigaScience* **8**, (2018).

12. Barnett, D. W., Garrison, E. K., Quinlan, A. R., Stř̈mberg, M. P. & Marth, G. T. Bamtools: A C++ API and toolkit for analyzing and managing BAM files. *Bioinformatics* **27**, 1691–1692 (2011).
13. Rhie, A., Walenz, B. P., Koren, S. & Phillippy, A. M. Merqury: Reference-free quality, completeness, and phasing assessment for genome assemblies. *Genome Biol.* **21**, (2020).
14. Kent, W. J. BLAT---The BLAST-Like Alignment Tool. *Genome Res.* **12**, 656–664 (2002).
15. Smit, A., Hubley, R. & Green, P. RepeatMasker Open-4.0. (2015).
16. Quinlan, A. R. & Hall, I. M. BEDTools: a flexible suite of utilities for comparing genomic features. *Bioinforma. Oxf. Engl.* **26**, 841–2 (2010).
17. Ruan, J. & Li, H. Fast and accurate long-read assembly with wtdbg2. *Nat. Methods* **17**, 155–158 (2020).
18. Chakraborty, M., Baldwin-Brown, J. G., Long, A. D. & Emerson, J. J. Contiguous and accurate de novo assembly of metazoan genomes with modest long read coverage. *Nucleic Acids Res.* **44**, gkw654–gkw654 (2016).
19. Marçais, G. *et al.* MUMmer4: A fast and versatile genome alignment system. *PLoS Comput. Biol.* **14**, e1005944–e1005944 (2018).
20. Chakraborty, M., Baldwin-Brown, J. G., Long, A. D. & Emerson, J. J. Contiguous and accurate de novo assembly of metazoan genomes with modest long read coverage. *Nucleic Acids Res.* **44**, gkw654 (2016).
