# Supplementary material for: A cattle graph genome incorporating global breed diversity
Source: Nat Commun. 2022 Feb 17;13:910. doi: 10.1038/s41467-022-28605-0 (PMC8854726; doi:10.1038/s41467-022-28605-0)
Supplement: Supplementary file 3 — Description of Additional Supplementary Files [file 41467_2022_28605_MOESM3_ESM.pdf]

Descriptions of additional supplementary data files

- Supplementary data 1 – Quality metrics for the N'Dama genome at the different stages of the assembly.
- Supplementary data 2 – Quality metrics for the Ankole genome at the different stages of the assembly.
- Supplementary data 3 – Motif enrichment analysis of the 20M high-quality novel sequences discovered from the 4 non-Hereford assemblies, using the five genomes as background, reporting the two-tailed binomial P- and Q-values.
- Supplementary data 4 – Putative novel genes discovered in the NOVEL sequence using the three approached described in the Materials and Methods (Augustus, Augustus on the sequences with 100bp flanking added and using BLASTX)
- Supplementary data 5 – Alignment metrics using bwa, a linear VG graph (VG1), a linear VG graph expanded with 11M variants from Dutta et al (2020; VG1p), a linear VG graph expanded with the 700K FORGe-prioritized variants (VG1f), a CACTUS-derived graph with 5 assemblies (VG5) and using a CACTUS-derived graph with 5 assemblies expanded with the 11M variants from Dutta et al. (2020; VG5p).
- Supplementary data 6 – List of genes predicted by Augustus and histogram of their sizes.
