# Supplementary material for: A cattle graph genome incorporating global breed diversity
Source: Nat Commun. 2022 Feb 17;13:910. doi: 10.1038/s41467-022-28605-0 (PMC8854726; doi:10.1038/s41467-022-28605-0)
Supplement: Supplementary file 10 — Reporting Summary [file 41467_2022_28605_MOESM10_ESM.pdf]

nature research

Corresponding author(s): James Prendergast

Last updated by author(s): November 30, 2021

Reporting Summary

Nature Research wishes to improve the reproducibility of the work that we publish. This form provides structure for consistency and transparency in reporting. For further information on Nature Research policies, see our Editorial Policies and the Editorial Policy Checklist.

Statistics

For all statistical analyses, confirm that the following items are present in the figure legend, table legend, main text, or Methods section.

n/a	Confirmed	
☐	☒	The exact sample size (*n*) for each experimental group/condition, given as a discrete number and unit of measurement
☐	☒	A statement on whether measurements were taken from distinct samples or whether the same sample was measured repeatedly
☐	☒	The statistical test(s) used AND whether they are one- or two-sided<br>*Only common tests should be described solely by name; describe more complex techniques in the Methods section.*
☐	☒	A description of all covariates tested
☐	☒	A description of any assumptions or corrections, such as tests of normality and adjustment for multiple comparisons
☐	☒	A full description of the statistical parameters including central tendency (e.g. means) or other basic estimates (e.g. regression coefficient) AND variation (e.g. standard deviation) or associated estimates of uncertainty (e.g. confidence intervals)
☐	☒	For null hypothesis testing, the test statistic (e.g. *F*, *t*, *r*) with confidence intervals, effect sizes, degrees of freedom and *P* value noted<br>*Give P values as exact values whenever suitable.*
☒	☐	For Bayesian analysis, information on the choice of priors and Markov chain Monte Carlo settings
☒	☐	For hierarchical and complex designs, identification of the appropriate level for tests and full reporting of outcomes
☒	☐	Estimates of effect sizes (e.g. Cohen's *d*, Pearson's *r*), indicating how they were calculated

Our web collection on statistics for biologists contains articles on many of the points above.

Software and code

Policy information about availability of computer code

Data collection	Publicly available data were retrieved from the databases using wget (v1.14).
Data analysis	The custom code for the study is available online on GitHub (https://github.com/evotools/CattleGraphGenomePaper).<br>Remaining software used include:<br>R (v3.5.3)<br>bedtools (v.2.30.0)<br>bcftools (v1.10)<br>vcftools (v0.1.13)<br>vg (v1.20.0 to 1.22.0)<br>samtools (v1.10)<br>Sambamba (v0.5.9)<br>Bowtie (v2.3.1)<br>GATK (v4.0.11.0)<br>FreeBayes (v1.3.1-16-g85d7bfc-dirty)<br>Deeptools (v3.5.1)<br>Genrich (v0.5)<br>bwa (v0.7.17)<br>delly (v0.8.5)<br>libbdsg (v0.3)<br>nextflow (v21.04)<br>bamtools (v2.4.2)<br>cactus (v2019.03.01)

CANU (v1.8)
WTDBG2 (v2.3)
wtpoa-cns (v2.3)
FALCON (v1.2.5)
Pilon (v1.23)
racon (v1.4.3)
minimap2 (v2.16-r922)
SibeliaZ (v1.1.0)
Ragout2 (v2.1.1)
Bionano Solve (v3.3_10252018)
RefAligner (v7915.7989rel)
LR_GapCloser (v1.1),
BUSCO (v3.0.2)
QUAST (v5.0.2)
merqury (v1.1)
meryl (v1.2)
FRC_Align (v1.3.0)
BlobToolKit (v2.3.3)
quickmerge (v0.3)
hal2vg (v2.1)
DustMasker (v1.0.0 from blast 2.9.0)
WindowMasker (v1.0.0 from blast 2.9.0)
RepeatMasker (v4.0.9)
trf (v4.09)
mash (v2.2)
HOMER (v4.10.4)
Augustus (v3.3.3)
DIAMOND (V2.0.6)
SURVIVOR (v1.0.7)
vcflib (v1.0.1)
trim_galore (v0.6.3)
AliTV (v1.0.6)
SuperExactTest (v1.0.7)
FORGe (v1.1.1)
SnpSift (v4.3t, build 2017-11-24 10:18)

For manuscripts utilizing custom algorithms or software that are central to the research but not yet described in published literature, software must be made available to editors and reviewers. We strongly encourage code deposition in a community repository (e.g. GitHub). See the Nature Research guidelines for submitting code & software for further information.

Data

Policy information about availability of data

All manuscripts must include a data availability statement. This statement should provide the following information, where applicable:

- Accession codes, unique identifiers, or web links for publicly available datasets
- A list of figures that have associated raw data
- A description of any restrictions on data availability

The long reads and short read data for the Ankole assembly have been deposited in the ENA database under project accession code (PRJEB39282)[https://www.ebi.ac.uk/ena/browser/view/PRJEB39282]. The long read and short reads data for the N'Dama sample have been deposited in the ENA database under project accessions codes (PRJEB39330)[https://www.ebi.ac.uk/ena/browser/view/PRJEB39330] and (PRJEB39334)[https://www.ebi.ac.uk/ena/browser/view/PRJEB39334]. The short read sequencing for the three Sahiwal and the three N'Dama samples have been deposited in the ENA database under project accessions codes (PRJEB39352)[https://www.ebi.ac.uk/ena/browser/view/PRJEB39352] and (PRJEB39353)[https://www.ebi.ac.uk/ena/browser/view/PRJEB39353], respectively. The N'Dama (GCA_905123515) and Ankole (GCA_905123885) assemblies have been deposited in the ENA database under accession codes (PRJEB41519)[https://www.ebi.ac.uk/ena/browser/view/PRJEB41519] and (PRJEB41564)[https://www.ebi.ac.uk/ena/browser/view/PRJEB41564], respectively. The optical mapping reads for the two N'Dama samples have been deposited in the ENA database under accession code (PRJEB47998)[https://www.ebi.ac.uk/ena/browser/view/PRJEB47998]. The ATAC-seq reads have been deposited in the ENA database under accession code (PRJEB49075)[https://www.ebi.ac.uk/ena/browser/view/PRJEB49075]. Output for the analyses can be visualised in (BOmA)[www.bomabrowser.com/cattle]. Source data are provided with this paper. Finally, the five VG graphs (VG1, VG1f, VG1p, VG5 and VG5p) as well as the CACTUS five-way whole genome alignments have been uploaded on datashare Zenodo with doi: 10.5281/zenodo.5749842 and 10.5281/zenodo.5750390. Source data for figures 1, 4B-D, 6A and Supplementary Figure 1 are provided with this paper.

Field-specific reporting

Please select the one below that is the best fit for your research. If you are not sure, read the appropriate sections before making your selection.

☒ Life sciences ☐ Behavioural & social sciences ☐ Ecological, evolutionary & environmental sciences

For a reference copy of the document with all sections, see nature.com/documents/nr-reporting-summary-flat.pdf

Life sciences study design

All studies must disclose on these points even when the disclosure is negative.

Sample size	Not applicable as generating genomes can be performed only on single individual genomes.
Data exclusions	No exclusions
Replication	Identification of structural variants from the genome graph was replicated using optical mapping data and comparisons to results from the Delly software
Randomization	Not relevant as generating reference genomes, which are based on single individual data.
Blinding	Blinding not relevant as need to know the breed being sampled

Reporting for specific materials, systems and methods

We require information from authors about some types of materials, experimental systems and methods used in many studies. Here, indicate whether each material, system or method listed is relevant to your study. If you are not sure if a list item applies to your research, read the appropriate section before selecting a response.

Materials & experimental systems

n/a	Involved in the study	
[ ]	[x]	Antibodies
[ ]	[x]	Eukaryotic cell lines
[x]	[ ]	Palaeontology and archaeology
[ ]	[x]	Animals and other organisms
[x]	[ ]	Human research participants
[x]	[ ]	Clinical data
[x]	[ ]	Dual use research of concern

Methods

n/a	Involved in the study	
[x]	[ ]	ChIP-seq
[x]	[ ]	Flow cytometry
[x]	[ ]	MRI-based neuroimaging

Antibodies

Antibodies used	anti-bovine SIRPα mono-clonal antibody (ILA-24), 1ug/ml
Validation	See Ellis et al. 1987 (PMID: 2963429)

Eukaryotic cell lines

Policy information about cell lines

Cell line source(s)	mouse mastocytoma cell line P815
Authentication	Just used as a spike-in. Reads were validated as aligning to the mouse genome.
Mycoplasma contamination	No cell lines were authenticated for Mycoplasma since irrelevant for the spike-in analysis.
Commonly misidentified lines (See ICLAC register)	The precise mouse line was irrelevant to this study as being used just as a spike-in

Animals and other organisms

Policy information about studies involving animals; ARRIVE guidelines recommended for reporting animal research

Laboratory animals	No laboratory animals were involved in the study
Wild animals	No wild animals were involved in the study
Field-collected samples	Samples were collected from farms in the relevant countries, identified by the local institutional partners. Only minimal amounts of blood were sampled from each animal which were then immediately returned to the herd
Ethics oversight	All protocols involving animals were approved prior to sampling by the relevant institutional animal care and use committee (ILRI IACUC or Roslin Institute Animal Welfare Ethical Review Body)

Note that full information on the approval of the study protocol must also be provided in the manuscript.
